# Supplementary material for: Population size, habitat association, and local residents’ attitude towards rock hyrax (Procavia capensis) in Zegie Peninsula, Ethiopia
Source: PLoS One. 2025 Jun 30;20(6):e0323878. doi: 10.1371/journal.pone.0323878 (PMC12208434; doi:10.1371/journal.pone.0323878)
Supplement: S1 File — (DOCX) [file pone.0323878.s001.docx]

PONE-D-24-32415R2

Population size, habitat association, and local residents’ attitude towards rock hyrax (*Procavia capensis*) in Zegie Peninsula, Ethiopia.

I have addressed the requested issues in the following ways:

1) The population data underlying the mean and other measures are presented in Table 1.

2) The following values were used to construct the graph (Fig. 1).

|  | Primary | Secondary | Higher education level | Religion | No education |
| --- | --- | --- | --- | --- | --- |
| Agree | 13 | 26 | 17 | 13 | 4 |
| Disagree | 30 | 4 | 2 | 39 | 55 |

3) This manuscript does not contain any images.
