# Supplementary material for: Population size, habitat association, and local residents’ attitude towards rock hyrax (Procavia capensis) in Zegie Peninsula, Ethiopia
Source: PLoS One. 2025 Jun 30;20(6):e0323878. doi: 10.1371/journal.pone.0323878 (PMC12208434; doi:10.1371/journal.pone.0323878)

PLOS ONE

Population size, habitat association and local residents' attitude towards rock hyrax (Procavia capensis) in Zegie Peninsula, Ethiopia

--Manuscript Draft--

**Manuscript Number:**	PONE-D-24-32415
**Article Type:**	Research Article
**Full Title:**	Population size, habitat association and local residents' attitude towards rock hyrax (Procavia capensis) in Zegie Peninsula, Ethiopia
**Short Title:**	Population size and local residents' attitude towards rock hyrax in Zegie Peninsula, Ethiopia
**Corresponding Author:**	Zewdu Kifle, Ph.D<br>Bahir Dar University College of Science<br>Bahir Dar, ETHIOPIA
**Keywords:**	Conservation, population estimation, residents' attitude, rock hyrax, traditional medicine, Zegie Peninsula
**Abstract:**	Given the current rate of habitat loss particularly in the tropics, reliable data on the population size and habitats of wild animals is crucial to initiate conservation and management activities in an area. Wildlife ecologists have not studied the ecology of most medium-sized mammals well. Rock hyrax (Procavia capensis) is one of the medium-sized mammals that are not well-studied in Ethiopia. Therefore, we conducted this study to determine the population size, and to examine the residents' attitude towards the rock hyrax in the Zegie Peninsula, Ethiopia. We applied the strip transect counting method to estimate the population size and determine the density of rock hyrax in the area. We used structured questionnaires and informant interviews to examine the attitude of local people towards rock hyrax in the region. We counted an average total population size of 469 individuals of rock hyraxes in the study area. Out of this population, adults comprised 72.4% and juveniles comprised 27.6%. Through extrapolation, we estimated the total population of c. 2184 rock hyraxes within the Zegie Peninsula. We counted 426 individuals of rock hyrax during the dry season while we counted 512 individual during the wet season. The average density of rock hyrax was 36.5 individuals/km2. The average juvenile-to-adult age ratio was 1:2.6 in the region. Most rock hyraxes were found in the lakeshore habitat followed by human residence areas. Most respondents had negative attitudes towards the rock hyraxes. Respondents reported that rock hyraxes caused pollution in the church and resident compounds due to their fecal pellets and urine. The respondents traditionally believed that rock hyraxes have a magical power that takes food from the local people and has destructive damage to the fruits and vegetables they harvest. Most respondents reported that rock hyraxes' body parts are used for the preparation of traditional medicines to cure human and cattle ailments. Key informants also reported that rock hyraxes' body parts are used to treat cattle anthrax, and their fecal pellets are used to treat leprosy. Most respondents did not support the conservation of rock hyraxes in the area. Our results provide a baseline for the development of conservation management strategies for the long-term survival of rock hyraxes in the region.
**Order of Authors:**	Zewdu Kifle, Ph.D
	Birkie Alehegn, Msc
**Additional Information:**	
**Question**	**Response**
**Financial Disclosure**<br><br>Enter a financial disclosure statement that describes the sources of funding for the work included in this submission. Review the submission guidelines for detailed	Yes

Powered by Editorial Manager® and ProduXion Manager® from Aries Systems Corporation

requirements. View published research articles from *PLOS ONE* for specific examples.<br><br>This statement is required for submission and **will appear in the published article** if the submission is accepted. Please make sure it is accurate.<br><br>**Funded studies**<br>Enter a statement with the following details:<br>• Initials of the authors who received each award<br>• Grant numbers awarded to each author<br>• The full name of each funder<br>• URL of each funder website<br>• Did the sponsors or funders play any role in the study design, data collection and analysis, decision to publish, or preparation of the manuscript?<br><br>Did you receive funding for this work?	
Please add funding details.<br>as follow-up to "**Financial Disclosure**<br><br>Enter a financial disclosure statement that describes the sources of funding for the work included in this submission. Review the submission guidelines for detailed requirements. View published research articles from *PLOS ONE* for specific examples.<br><br>This statement is required for submission and **will appear in the published article** if the submission is accepted. Please make sure it is accurate.	Bahir Dar University

**Funded studies**<br>Enter a statement with the following details:<br>• Initials of the authors who received each award<br>• Grant numbers awarded to each author<br>• The full name of each funder<br>• URL of each funder website<br>• Did the sponsors or funders play any role in the study design, data collection and analysis, decision to publish, or preparation of the manuscript?<br><br>Did you receive funding for this work?"	
Please select the country of your main research funder (please select carefully as in some cases this is used in fee calculation).<br>as follow-up to "**Financial Disclosure**<br><br>Enter a financial disclosure statement that describes the sources of funding for the work included in this submission. Review the submission guidelines for detailed requirements. View published research articles from *PLOS ONE* for specific examples.<br><br>This statement is required for submission and **will appear in the published article** if the submission is accepted. Please make sure it is accurate.<br><br>**Funded studies**<br>Enter a statement with the following details:<br>• Initials of the authors who received each award<br>• Grant numbers awarded to each author<br>• The full name of each funder<br>• URL of each funder website<br>• Did the sponsors or funders play any role in the study design, data collection and analysis, decision to publish, or preparation of the manuscript?<br><br>Did you receive funding for this work?"	ETHIOPIA - ET
**Competing Interests**	The authors have declared that no competing interests exist.

Use the instructions below to enter a competing interest statement for this submission. On behalf of all authors, disclose any competing interests that could be perceived to bias this work—acknowledging all financial support and any other relevant financial or non-financial competing interests.<br><br>This statement is required for submission and **will appear in the published article** if the submission is accepted. Please make sure it is accurate and that any funding sources listed in your Funding Information later in the submission form are also declared in your Financial Disclosure statement.<br><br>View published research articles from *PLOS ONE* for specific examples.<br><br>**NO authors have competing interests**<br><br>Enter: *The authors have declared that no competing interests exist.*<br><br>**Authors with competing interests**<br><br>Enter competing interest details beginning with this statement:<br><br>*I have read the journal's policy and the authors of this manuscript have the following competing interests: [insert competing interests here]*<br><br>* typeset	
**Ethics Statement**<br><br>Enter an ethics statement for this submission. This statement is required if the study involved:<br><br>• Human participants<br>• Human specimens or tissue<br>• Vertebrate animals or cephalopods<br>• Vertebrate embryos or tissues	This study was carried out according to the guidelines of the review board of Bahir Dar University (BDU) of the human ethics research. The interviewer fully informed to the respondents on the aim of the research and how the data obtained from them would be used. Confidentiality of information was assured by the investigator. Participants gave their consent verbally after being explained the aim of the survey and how the interview would proceed. Consent was obtained by having participants state that they agree to participate.

Powered by Editorial Manager® and ProduXion Manager® from Aries Systems Corporation

• Field research<br><br>Write "N/A" if the submission does not require an ethics statement.<br><br>General guidance is provided below. Consult the submission guidelines for detailed instructions. **Make sure that all information entered here is included in the Methods section of the manuscript.**	

**Format for specific study types**<br><br>**Human Subject Research (involving human participants and/or tissue)**<br>• Give the name of the institutional review board or ethics committee that approved the study<br>• Include the approval number and/or a statement indicating approval of this research<br>• Indicate the form of consent obtained (written/oral) or the reason that consent was not obtained (e.g. the data were analyzed anonymously)<br><br>**Animal Research (involving vertebrate animals, embryos or tissues)**<br>• Provide the name of the Institutional Animal Care and Use Committee (IACUC) or other relevant ethics board that reviewed the study protocol, and indicate whether they approved this research or granted a formal waiver of ethical approval<br>• Include an approval number if one was obtained<br>• If the study involved *non-human primates*, add *additional details* about animal welfare and steps taken to ameliorate suffering<br>• If anesthesia, euthanasia, or any kind of animal sacrifice is part of the study, include briefly which substances and/or methods were applied<br><br>**Field Research**<br><br>Include the following details if this study involves the collection of plant, animal, or other materials from a natural setting:<br>• Field permit number<br>• Name of the institution or relevant body that granted permission	
**Data Availability**<br><br>Authors are required to make all data underlying the findings described fully available, without restriction, and from the time of publication. PLOS allows rare exceptions to address legal and ethical concerns. See the PLOS Data Policy and FAQ for detailed information.	Yes - all data are fully available without restriction

A Data Availability Statement describing where the data can be found is required at submission. Your answers to this question constitute the Data Availability Statement and **will be published in the article**, if accepted.<br><br>**Important:** Stating 'data available on request from the author' is not sufficient. If your data are only available upon request, select 'No' for the first question and explain your exceptional situation in the text box.<br><br>Do the authors confirm that all data underlying the findings described in their manuscript are fully available without restriction?	
**Describe where the data may be found in full sentences. If you are copying our sample text, replace any instances of XXX with the appropriate details.**<br><br>• If the data are **held or will be held in a public repository**, include URLs, accession numbers or DOIs. If this information will only be available after acceptance, indicate this by ticking the box below. For example: *All XXX files are available from the XXX database (accession number(s) XXX, XXX.*).<br>• If the data are all contained **within the manuscript and/or Supporting Information files**, enter the following: *All relevant data are within the manuscript and its Supporting Information files.*<br>• If neither of these applies but you are able to provide **details of access elsewhere**, with or without limitations, please do so. For example:<br><br>*Data cannot be shared publicly because of [XXX]. Data are available from the XXX Institutional Data Access / Ethics Committee (contact via XXX) for researchers who meet the criteria for access to confidential data.*<br><br>*The data underlying the results presented in the study are available from (include the name of the third party*	All relevant data are within the manuscript and its Supporting Information files.

and contact information or URL).<br>• This text is appropriate if the data are owned by a third party and authors do not have permission to share the data.<br><br>* typeset	
Additional data availability information:	Tick here if the URLs/accession numbers/DOIs will be available only after acceptance of the manuscript for publication so that we can ensure their inclusion before publication.

Powered by Editorial Manager® and ProduXion Manager® from Aries Systems Corporation

Manuscript

Click here to access/download;Manuscript;Zewdu Kifle_Population size, of rock hyrax.docx

Population size, habitat association and local residents' attitude towards rock hyrax (*Procavia capensis*) in Zegie Peninsula, Ethiopia

Birkie Alehegn[1], Zewdu Kifle*[1]

[1]Department of Biology, Bahir Dar University, Bahir Dar, Ethiopia

Corresponding author: Zewdu Kifle, email: zewdu96@yahoo.com;

ORCID: https://orcid.org/0000-0002-0014-3525

ABSTRACT

Given the current rate of habitat loss particularly in the tropics, reliable data on the population size and habitats of wild animals is crucial to initiate conservation and management activities in an area. Wildlife ecologists have not studied the ecology of most medium-sized mammals well. Rock hyrax (*Procavia capensis*) is one of the medium-sized mammals that are not well-studied in Ethiopia. Therefore, we conducted this study to determine the population size, and to examine the residents' attitude towards the rock hyrax in the Zegie Peninsula, Ethiopia. We applied the strip transect counting method to estimate the population size and determine the density of rock hyrax in the area. We used structured questionnaires and informant interviews to examine the attitude of local people towards rock hyrax in the region. We counted an average total population size of 469 individuals of rock hyraxes in the study area. Out of this population, adults comprised 72.4% and juveniles comprised 27.6%. Through extrapolation, we estimated the total population of c. 2184 rock hyraxes within the Zegie Peninsula. We counted 426 individuals of rock hyrax during the dry season while we counted 512 individual during the wet season. The average density of rock hyrax was 36.5 individuals/km^2. The average juvenile-to-adult age ratio was 1:2.6 in the region. Most rock hyraxes were found in the lakeshore habitat followed by human residence areas. Most respondents had negative attitudes towards the rock hyraxes. Respondents reported that rock hyraxes caused pollution in the church and resident compounds due to their fecal pellets and urine. The respondents traditionally believed that rock hyraxes have a magical power that takes food from the local people and has destructive damage to the fruits and vegetables they harvest. Most respondents reported that rock hyraxes' body parts are used for the preparation of traditional medicines to cure human and cattle ailments. Key informants also reported that rock hyraxes' body parts are used to treat cattle anthrax, and their fecal pellets are

used to treat leprosy. Most respondents did not support the conservation of rock hyraxes in the area. Our results provide a baseline for the development of conservation management strategies for the long-term survival of rock hyraxes in the region.

Keywords: Conservation, population estimation, residents’ attitude, rock hyrax, traditional medicine, Zegie Peninsula

Introduction

The biological diversity around the globe represents a resource that offers benefits to current and future generations [1]. However, because of the need to feed a growing human population, forests have been cut down for agriculture, livestock grazing, and urbanization [2, 3]. This has led to the fragmentation of the landscape and has altered the distribution and abundance of many wild animals and plants [4]. The effects and magnitude of fragmentation on biodiversity are complex [4, 5]. The human population in Africa is growing at a rapid rate which needs space to live and harvest. Human interference in the form of clearing animal habitats for agriculture has impacted African wildlife [6].

Ethiopia possesses different altitude ranges and topography [7]. The altitudinal variation of the country ranges from 126 meters below sea level in the Danakil Depression of the Afar region to the highest peak of 4,620 meters above sea level on Mount Ras Dejen of the Simien Mountains National Park. These altitude and topography variation creates diverse climatic conditions varying from the hot and dry desert in the lowland areas to cold and humid alpine habitats in the highlands. These factors also strongly influence Ethiopia’s extraordinary range of terrestrial and aquatic ecosystems which have contributed to high flora and fauna diversity and rate of endemism [8]. Past geological history and wide-ranging climatic features have made Ethiopia the home of diverse endemic fauna and flora [9].

Rock hyrax looks like a large guinea pig or a rabbit with rounded, short ears and no visible tail. The rock hyrax’s coat ranges in color from yellowish to grayish-brown, with a lighter underbelly. They have short legs very long bodies that are concealed by a hunched posture and long fur [10]. They have long, tactile hairs, especially on the muzzle, throat, cheeks, and rump. Rock hyraxes eat mostly grass, fruits, and leaves [10, 11]. They have disproportionately large jaws and an ability to draw the lips right back allowing them to bite off and chew large mouthfuls of vegetation at a time. Their habitats include the savanna, shrubland, desert, and

rocky areas, such as cliffs and mountain peaks [10]. Being, rock hyraxes are slow-moving and with only a few defensive mechanisms against predators, they depend on rocky outcrops, which provide protection from predators, access to safe foraging areas, and good vantage points [12].

Hyraxes are medium-sized terrestrial mammals native to Africa and the Middle East. Hyrax comprises three extant genera: *Procavia*, *Heterohyrax*, and *Dendrohyrax*. Five extant species of hyraxes are recognized: the rock hyrax (*Procavia capensis*) and the yellow-spotted rock hyrax (*Heterohyrax brucei*), which both live on rock outcrops, including cliffs in Ethiopia and isolated granite outcrops called koppies in southern Africa; the western tree hyrax (*Dendrohyrax dorsalis*), southern tree hyrax (*D. arboreus*), and eastern tree hyrax (*D. validus*). Their distribution is limited to Africa, except for *P. capensis*, which is also found in the Middle East

In Ethiopia, rock hyrax (*P. capensis*) lives in rock outcrops, piles of boulders, and fractured cliff faces [13]. Rock hyrax has the widest geographical and altitudinal distribution among the hyraxes [14]. Even if there are no major threats to rock hyrax, predation, intra-specific competition, immigration, territorial fighting and dispersal regulate rock hyrax population. Rock hyraxes are hunted by people for their meat and soft fur. For example, the Hadza or Watindiga, a bushman tribe in Tanzania, hunt rock hyraxes for food [15]. In some areas of Egypt, it is hunted for food [16]. In countries like Kenya and Israel, the rock hyrax is not supported to live in their locality for being a reservoir of cutaneous *leishmaniasis* [17].

Rock hyrax is classified as a species of Least Concern Red List of Threatened Species [18] However; their populations have declined due to habitat loss, disease, and predation [19]. They are considered as a pest in some areas, competing with farmers by eating their crops. They are also subjected to threats from various avian and mammalian predators.

Assessing the population size of wild animals and their habitat association as well as understanding the attitudes of the local people towards the wild animal is important for designing sustainable conservation and management strategies [20]. Only a few studies have been conducted on the population size, distribution, and ecology of rock hyrax in Ethiopia [21, 22, 23]. Thus, the population size and distribution of rock hyrax is poorly known in Ethiopia. Specifically, there is no information on the population size of rock hyrax in the Zegie Peninsula, Ethiopia. In addition, the attitude of local people towards rock hyrax has not been documented in Ethiopia. Therefore, this study is the first and the objectives of the study were (1) to determine the population size of rock hyrax in the Zegie Peninsula**;** (2) to determine the habitat association

of rock hyrax in the area; and (3) to examine the attitude of local people towards rock hyrax conservation. This study will be fundamental for further studies (e.g., traditional medicine of rock hyrax and other ecological and behavioral aspects) in the region.

Materials and Methods

Ethical considerations

This study was carried out according to the guidelines of the review board of Bahir Dar University (BDU) of the human ethics research. The interviewer fully informed to the respondents on the aim of the research and how the data obtained from them would be used. Confidentiality of information was assured by the investigator. Participants gave their consent verbally after being explained the aim of the survey and how the interview would proceed. Consent was obtained by having participants state that they agree to participate.

Study area

We carried out this study in the Zegie Peninsula, the southwestern shore of Lake Tana, Ethiopia (Fig 1). Zegie Peninsula covers 13.47 Km^2. The Zegie Peninsula is situated at 11°40’ to 11°43’ N latitude and 37°19’ to 37°21’ E. It is found at elevations between 1775 m and 1985 m above sea level. Zegie Peninsula is home to many Ethiopian Orthodox churches and monasteries dating back to the 13th century with a unique cultural, and religious heritage, and historical and aesthetic value. It is an important site for persevering religion and culture. It is one of the tourist destination sites in Ethiopia. The climate of Zegie Peninsula is a warm-temperate tropical highland monsoon with a mean temperature of 21.9°C. The area experiences two main seasons: the wet season and the dry season. The wet season typically occurs between June and October, while the dry season occurs between November and May.

The Peninsula is surrounded by Lake Tana in the northern and eastern directions, wetlands in its western parts, and farmlands in the southern directions. The dominant vegetation in this habitat are *Mimusops kumme*, *Syzygium guineense*, *Ficus vasta*, Shenbeko, *Jasminum grandiflorum*, *Momordica foetida*, *Clematis simensis*, *Tragia brevipes*, *Urera hypselodendron and Phytolacca dodecandra*. The Zegie Peninsula is home to different mammalian species like leopard (*Panthera pardus*), grivet monkey (*Chlorocebus aethiops*), rock hyrax (*Procavia capensis*), bushpig (*Potamochoerus larvatus*), duiker (*Sylvicapra grimmia*), and common

bushbuck (*Tragelaphus scriptus*). In addition, the peninsula supports diverse species of birds, amphibians, reptiles, mollusks, and insects. However, its wild animals and their habitats are under threat due to deforestation and illegal hunting practices [24]. For example, bushbuck and warthog have been eradicated due to illegal hunting in the area [25].

Population size and habitat association of rock hyraxes

Between January 10, 2021 and June 15, 2021, we applied a line transect method to estimate the population size of rock hyrax in the study area. The census zone was divided into several sample units. The selection of the sampling units was done by using two-stage sampling techniques. We divided the study area into four habitat strata: The lakeshore stratum of the coffee-dominated semi-natural forest, human premises, tall trees with coffee plantations, and bushland. We established a total of 18 transect lines in these four habitats. The distance between two consecutive transect lines was 200 to 300 m to avoid double counting of rock hyrax. The length and the number of transects were proportional to the size of the habitat type. During the transect survey, we recorded the number of rock hyrax seen, the age category, and the habitat type. We conducted transect walks for three consecutive days per month from 3:30-5:30 hours in the morning and from 8:30-11:30 hours in the late afternoon. We surveyed transects by direct counting method walking at an average speed of 1km/hr [26]. Within the line transect, we moved along the lines, looking for the target species with a 25 m fixed transect width to the left and right sides of the line [27]. We sampled 2.71 km^2 from the total size of 12.62 km^2 area. The study covered dry (Feb, Mar, and Apr) and wet (Jun, Jul, and Sep) seasons.

Questionnaire survey

Between March 16, 2021 and June 20, 2021, we used a structured questionnaire interview method to collect data on the attitude of local people towards rock hyrax. We applied a two-stage sampling technique to select the respondents. First, we selected seven villages from two *kebeles* (i.e., Ura and Yiganda-Mehale Zegie) using purposive sampling. Second, we selected the household heads (respondents) from the sampling villages by systematic random sampling method using the recorded book of each village found in the *kebele* (local office). We interviewed a total of 210 respondents out of 1,116 households inhabiting the Peninsula. We designed the structured questionnaire to examine the attitudes, practices, and beliefs of the local

people, knowledge of its significance on rock hyrax as well as to identify factors that influence community perceptions on rock hyraxes. The questionnaire also included respondents' level of education, age, sex, educational status, and livelihood. To triangulate the data collected by questionnaire, we undertook key informant interviews with nine dwellers (community leaders, elders, religious leaders, and association leaders) to express the culture and practice related to rock hyrax in the study area.

Data analysis

We summed up all transects of each habitat results to determine the total population of rock hyraxes [28]. We extrapolated the transect result to estimate the total population number of rock hyrax within the study area. We excluded bushland habitat from the analysis since we did not record rock hyrax in this habitat during the study period. We determined the density of the rock hyrax population by dividing the counted population (individuals) in the sample sites by the total area of the study area (km^2). We analyzed data using descriptive statistics. We used ANOVA to examine the population size difference of rock hyraxes between the dry and wet seasons. We used the Chi-square test to examine the attitude of local people towards rock hyrax based on respondents' educational background and sex. The level of rejection for all tests set at $\alpha = 0.05$.

Results

Population size of rock hyrax

We counted a total mean of 469 rock hyrax individuals in the Zegie Peninsula. Out of this population, the number of adults was 339.5 (72.4%) and 129.5 (27.6%) were juveniles. The average adult-to-juvenile age ratio was 1:2.6. Through extrapolation, we estimated the total population size of 2184 individuals of rock hyrax in the Zegie Peninsula. The density of rock hyrax was 37.2 individuals/km^2 in the Peninsula.

From the total population, we counted 426 individuals of rock hyrax during the dry season and 512 during the wet season (Table 1). The population size of rock hyrax did not show a significant difference between the wet and dry seasons ($F_{1,6}$ = 5.988, P = 0.068). During the dry season, adults constituted 75.1% while 24.9 % were babies (juveniles) while during the wet season, adults constituted 70.1% and 29.9% were babies. Adult to juvenile ratio was 1:3.0 during the dry season while the ratio was 1:2.3 during the wet season. The density of rock hyrax during the dry season was 33 individuals/km^2, and it was 40 individuals/km2 during the wet season.

We recorded the highest numbers of rock hyrax in the lakeshore (223.5, 47.7%) while we recorded the lowest numbers of rock hyrax in the forest habitat (35.5, 7.6%). We didn't record rock hyrax in the bushland habitats. We recorded the highest population size of rock hyraxes in the lakeshore habitat during the dry (210 individuals) and the wet (237 individuals) seasons.

Questionnaire survey

Socio-demographic features of the respondents

We conducted the questionnaire interviews with 203 respondents of which 105 were males and 98 were females with ages ranging from 21 to 65 years (Table 2). Most (97.5%) respondents reported that their livelihood depends on a coffee plantation, fruit production, fishing, wood (log) selling for firewood, small-scale trading, handcraft making, and selling, Most (97.0%) respondents had their farmlands. Most (71.4%) respondents did not have sheep while a few (28.6%) respondents had sheep. Few (2.5%) respondents were governmental and non-governmental employees. Many (29.0%) respondents had not attended both formal and informal education.

Local residents' attitudes towards rock hyraxes

More than half (57.6%) of respondents had negative attitudes towards rock hyraxes. On the contrary, other (42.4%) respondents had positive attitudes towards rock hyraxes in their presence in the region (Table 3). Male respondents had a better feeling about the presence of rock hyraxes than female respondents, and the difference was significant ($\chi2 = 27.70$, df = 1, $P = 0.001$). Higher educational background respondents had a better feeling about the presence of rock hyraxes than uneducated and religious education respondents and the difference was significant ($\chi2 = 90.91$, df = 4, $P = 0.001$).

Most (64.5%) respondents disagreed with the idea of rock hyrax conservation in the region, while other (35.5%) respondents agreed with the idea of rock hyrax conservation. Most of the uneducated, religious, and primary educated respondents disagreed on rock hyraxes protection but many of secondary educated and above respondents agreed on the conservation of rock hyraxes in their localities (Fig 2).

Perceived costs and benefits of rock hyrax presence

Most (80.0%) respondents reported that rock hyraxes damaged fruits and vegetables. While a few (20%) respondents believed that rock hyraxes did not cause significant crop damage in their localities. Similarly, the key informant interview participants deeply expressed the destructive behavior of rock hyrax on crops. In addition, many respondents reported that economically important plants like *Rhamnus prinoides*, *Carica papaya*, *Citrus lemon*, *Eucalyptus camaldulesis*, *Mimusops kummel*, and *Acacia pilispina* are damaged by rock hyraxes.

Many local people had bad traditional myths about the rock hyraxes that they have some kind of magic power (locally known as "*wosaji*") i.e., rock hyraxes take food magically as they see people eating. In addition, the other common problem was the occurrence of rock hyraxes' feces and urine that created pollution (bad smell) in and around churches and resident house compounds.

Most (93%) respondents replied that rock hyraxes have some benefits. Of these respondents, 88.0% described that rock hyraxes' body parts are used for the preparation of traditional medicines to cure some human and cattle ailments. While, a few (3.0%) respondents reported that the skin of rock hyrax is used as traditional medicine (specific diseases were not mentioned), and a few (2.0%) respondents used rock hyraxes for their dog's food. Key informants also reported that rock hyraxes' flesh is used to treat cattle anthrax, and their fecal pellets are used to treat leprosy.

Awareness of rock hyrax population and conservation status

When asked about the population size of rock hyrax, 112 (55.0%) respondents reported that the population size had decreased in the region, 76 (37.0%) believed it had increased in recent years and 15 (8.0%) had not known about the number of rock hyrax whether they increased or decreased. Many (56.0%) respondents saw most frequently three individuals of rock hyraxes in a group and other respondents (44.0%) respondents saw four to five individuals in the group. Most of the key informant interviewees also informed that rock hyraxes were not seen as widely in lakeshores and in church compounds as they were seen years before. They reported that hunting for traditional medicine and dogs, habitat disturbance, and damage to shelters like big trees and stone fences are responsible for their decrement in the area.

Controlling mechanisms

Many (57.0%) respondents used killing and 43.0% of respondents used chasing as a means of controlling crop damage caused by rock hyraxes and avoiding pollution by its fecal pellets and urine. Local people killed rock hyrax with spears, poisons, traps, and dogs. They also chased away rock hyrax by throwing stone, chasing with dogs, and making noise.

Conservation threats to rock hyraxes

In the Zegie Peninsula, 115 (56.0%) respondents reported threats to rock hyraxes in their localities, while other (44.0%) respondents did not believe in the presence of threats to rock hyraxes in the region. Of the 115 respondents who reported threats to rock hyraxes, most (65.2%) respondents considered hunting and killing as the main threat followed by predation by dogs (28.8%) and 6.0% habitat disturbance.

Discussion

Most researchers do not study the population ecology and behavioral ecology of medium-sized mammals and their conflicts with local people. Our study investigated the population size of rock hyrax as well as the extent of human-rock hyrax conflict in the Zegie Peninsula, Ethiopia. We found an average of 469 rock hyrax populations in the peninsula. From this total population of rock hyrax adults constituted more proportion than the babies (juveniles). Similarly, [13] recorded more adult ratio than babies in the Bale Mountains National Park, Ethiopia. This might be due to the survival rate of adults more than young individuals.

In the present study, the average density of rock hyrax was 36.5 individuals/km2. On the contrary, [13] reported that the mean population density of 664.5 and 529.9 individuals/km2 during the wet and dry seasons in the Bale Mountain National Park. Rock hyrax population density studies elsewhere have revealed a density of 20 to 100 individuals/ km² in Mount Kenya [29], 500 to 4000 individuals/km2 in the Serengeti National Park, Tanzania [19], 73 and 94 individuals/ km² in the Matobo National Park, Zimbabwe [28]. The minimum availability of rock outcrops, cliffs with crevices, caves, and gorges having rock piles or crevices in the Zegie Peninsula might be the case for low population density in the region. In addition, high habitat disturbance in the current study area might be the cause for the minimum density of rock hyrax. Severe habitat alterations due to various human activities in the natural habitat influence the ecology and population dynamics of wild animals [30]. Natural habitat disturbance through human activities influence the population size and density of rock hyrax, highest population size recorded in areas where there are rocky outcrops, cliffs, and grasses and shrubs in the vicinity of their shelter [13].

[11] found the extreme population fluctuations of hyrax at Montopos in Zimbabwe and in the Serengeti of Tanzania. [31] in National Park, South Africa also found that hyrax populations fluctuate mainly due to predation, food availability, drought, and infectious disease. In the present study, we counted more rock hyrax populations during the wet season than in the dry season. This might be the birth of more pups during the wet season when the availability of food that can satisfy their nutritional demands is higher during this season. The wet season is full of more nutritious food items, which could promote the breeding of animals and birth. Hyrax population fluctuation between seasons is mainly due to predation, droughts, and infectious diseases [31, 32]. During the dry season, hyraxes expose themselves to greater predation risk as they seek food farther from cover and refuges [13, 31].

Rock hyraxes were not uniformly distributed in the study area. We counted more rock hyraxes around the lakeshore habitat. This might be due to the availability of save and abundant rocky outcrops in this habitat for rock hyraxes to forage and shelter. Similarly, [13] also documented that rock hyrax is not uniformly distributed in the Bale Mountains National Park, Ethiopia. The distribution of rock hyrax mainly depends on the availability of food and shelter that allows it to hide and watch its predators easily. [29] also documented that the distribution of rock hyraxes is delineated by the presence of appropriate shelter. Similarly, [33] reported that

rock hyraxes are typically associated with rocky outcrops, cliffs, or piles of boulders with bushes. [34] stated that the distribution of rock hyrax decreases around human premises due to habitat loss and human disturbance.

Most people did not like the presence of rock hyraxes in their locality. This might be due to their destruction of fruits and vegetables, causing pollution around churches and houses as they defecate or urinate, and the traditional belief of the people that it had magic power to take food they eat as it saw people when eating. Rock hyraxes are reservoirs of cutaneous *leishmaniasis* that can lead to disease outbreaks around human settlements [17, 35]. Members of rock hyrax colonies urinate and defecate in designated areas (latrines) that might cause bad smells in an area. This behavior of rock hyrax might not be confortable for local people and develop a negative attitude toward this animal.

In the present study, rock hyrax damages crops. When rock hyraxes inhabit rock boulders or cliffs near farmlands they heavily utilize crop seedlings [13. 21]. [36] also documented that, rock hyraxes inhabiting close to farmlands damage crops. Similar studies in different parts of Africa also revealed that wild animals posed major threats to crops and livestock [37]. Baboons in particular are important crop raiders across much of their ranges in Ethiopia [38, 39].

A strong attitudinal difference was seen towards rock hyrax presence related to educational background. Respondents of higher educational backgrounds had better feeling about the presence of rock hyraxes in their locality than uneducated and religious educational backgrounds. Moreover, males had a better feeling of the presence of rock hyraxes in their locality than females. Crop damage and livestock predation by wild animals influence the attitude of local farmers toward their conservation [38, 39, 40, 41].

Most illiterate and primarily educated respondents disagreed with the idea of rock hyrax conservation. This indicated that most of the dwellers did not have enough awareness and had less concern for rock hyrax protection. [42] noted that concerns about the species have been raised by the landowners in the Magaliesberg province, North West Region of South Africa. On the contrary, most of the secondary educated and above respondents agreed on the protection of rock hyraxes in the region. This showed that formal education is important to develop wildlife conservation initiatives in an area.

Conclusion

This study provides crucial information on the population size, habitat association of rock hyrax, and local peoples' attitude towards as well as the ethno-zoological importance of this wild animal inhabiting the Zegie Peninsula, Ethiopia. We identified the Peninsula as an important site for the conservation of rock hyrax. Human disturbances mainly with the absence of enough shelter in the area affect the population density of rock hyrax in the area.

Even if rock hyraxes damaged crops, local people use rock hyraxes as traditional medicine to treat human and livestock aliments. This practice is so important to promote conservation measures for rock hyrax in Ethiopia. The local administration and other governmental and non-governmental organizations should work cooperatively to create awareness and educate the local community about the economic and ecological importance and conservation of rock hyrax. This study provides baseline information for further traditional medicine of rock hyrax as well as ecological and behavioral aspects in the region.

Declaration of competing interest

The authors declare that he has no known competing financial interests or personal relationships that could have appeared to influence the work reported in this paper.

Acknowledgements

This work was funded by the Bahir Dar University. We would like to thank the Zegie district administers for allowing us to carry out this study in the Zegie Peninsula. Our thanks also go to the local people who answered the questionnaire survey politely.

References

1. Toledo VM. The biodiversity of Latin America: A threatened heritage. Ambiente y Desarrollo 1988; 4: 13–24.
2. Altieri MA, Toledo VM. The agroecological revolution in Latin America: Rescuing nature, ensuring food sovereignty and empowering peasants. J Peasant Stud. 2011; 38: 587–612.
3. Laurance W, Sayer J, Cassman K. Agricultural expansion and its impacts on tropical nature. Trends in Ecol Evol. 2014; 29: 107–116.
4. Fahrig L. Effects of habitat fragmentation on biodiversity. Annual Review of Ecology Evol Syst. 2003; 34: 487–515.

5. Pardini R, Souza SM, Braga-Neto R, Metzger JP. The role of forest structure, fragment size and corridors in maintaining small mammal abundance and diversity in an Atlantic forest landscape. Biol Conserv. 2005; 124: 253–266.
6. Harris JD, Brown PL. Wildlife; Destruction, Conservation and Biodiversity. Nova Science Publishers, 2009.
7. Husen A, Mishra V, Semwal K, Kumar D. Biodiversity Status in Ethiopia and Challenges. Environ. Pollut Biodiver. 2012; 1: 31–79.
8. Awas T. Plant Diversity in Western Ethiopia: Ecology, Ethnobotany and Conservation Department of Biology. Degree of Doctor of Philosophy. University of Oslo, Norway, 2007.
9. Yalden DW, Largen MJ. Endemic mammals of Ethiopia. Mammal Review. 2008; 22:115–150.
10. Kingdon J. The Kingdon Field Guide to African Mammals. Bloomsbury Publishing. 2015
11. Hoeck HN, Bloomer P. Rock Hyrax *Procavia capensis*. Bloomsbury, London. 2013.
12. Fairall N, Eloff AK, Mcnairn IS. Integration of metabolism and digestion in the hyrax. S Afr J Anim Sci 1983; 13: 79–80.
13. Teklehaimanot G. Population Ecology and Threats of the Rock Hyrax (*Procavia Capensis*) in Bale Mountains National Park, Ethiopia. Zoological Sciences. PhD thesis, Addis Ababa University. 2015.
14. Stuart C, Stuart T. Field Guide to the Larger Mammals of Africa. Struik Publishers, Cape Town. 2000.
15. Smith EA., Hill K, Marlowe FW, et al. Wealth transmission and inequality among hunter-gatherers. J curr Anthropol. 2010; 51: 1–16.
16. Nowak RM. Walker's Mammals of the World, The John Hopkins University Press, Baltimore, Maryland. 1999.
17. Jacobson R, Eisenberger C, Svobodova M, et al. Outbreak of Cutaneous Leishmaniasis in Northern Israel. J Infect Dis. 2003; 188: 1065–1073.
18. Butynski T, Hoeck H, Koren L, De Jong YA., *Heterohyrax brucei*. The IUCN Red List of Threatened Species. 2015; e.T41766A2128587
19. Hoeck H, Klein H, Hoeck P, Flexible Social Organization in Hyrax1). Zeitschrift für Tierpsychologie. 1982; 59: 265–298.

20. Newmark W, Manyanza D, Gamassa D.-G, Sariko H. The Conflict between Wildlife and Local People Living Adjacent to Protected Areas in Tanzania: Human Density as a Predictor. Conserv Biol. 1994: 8: 249–255.
21. Girma, T, Teshome Z, Mahari M, Knowledge, attitude and practices of peasants towards hyraxes in two selected church forests in Tigray Region, Northern Ethiopia. Int J Biodiv Conserv. 2015; 7: 299–307.
22. Asrat A. The Tree Hyrax (*Dendrohyrax arboreus*): Feeding Behaviour, Activity Patterns and Traditional Medicinal Use in Kafa Zone, Southwest Ethiopia. Masters thesis, Addis Ababa University. 2016.
23. Teklehaimanot G, Balakrishnan M, Distribution and Population Status of the Endemic Rock Hyrax Sub-species (Procavia capensis capillosa) of the Bale Mountains, Ethiopia. Int J Ecol Environ Sci. 2018; 44: 1–9.
24. Belay B, Zewdie S, Mekuria W, Abiyu A, Amare D, Woldemariam T. Woody species diversity and coffee production in remnant semi-natural dry Afromontane Forest in Zegie Peninsula, Ethiopia. Agrofor Syst. 2019; 93: 1793–1806.
25. Gebeyehu G, Bekele A. Human-wildlife conflict in Zegie Peninsula (Ethiopia) with emphasis on grivet monkey (*Cercopithecus aethiops aethiops*). SINET: Ethio. J. Sci. 2011; 32: 99–108.
26. Whittaker DG, Van Dyke WA, Love SL. Evaluation of Aerial Line Transect for Estimating Pronghorn Antelope Abundance in Low-Density Populations. Wildl Soc Bull. 2003; 31: 443–453.
27. Bowland A. Perrin M. Temporal and spatial patterns in blue duikers *Philatomba monticola* and red duikers *Cephalophus natalensis*. J Zool. 2009; 237, 487–498.
28. Barry R, Mundy P. Population dynamics of two species of hyraxes in the Matobo National Park, Zimbabwe. Afr J Ecol. 2002; 36: 221–233.
29. Young TP, Evans M.R. Alpine vertebrates of Mount Kenya, with particular notes on the Rock Hyrax. J East Afr Nat Hist. 1993; 82: 55–79.
30. Geist HJ. Lambin E.E. Proximate causes and underlying driving forces of tropical deforestation. BioScience, 2002; 52: 143–150.

31. Druce D, Brown J, Castley G, Kerley G, Kotler B, Slotow R, Knight M. Scale-dependent foraging costs: Habitat use by rock hyraxes (*Procavia capensis*) determined using giving-up densities. Oikos. 2006: 115: 513–525.
32. Parsons SDC, Smith SGD, Martins Q, et al. Pulmonary infection due to the dassie bacillus (*Mycobacterium tuberculosis* complex sp.) in a free-living dassie (rock hyrax - *Procavia capensis*) from South Africa. Tuberculosis, 2008; 88: 80–83.
33. Skinner JD, Chimimba CT. The Mammals of the Southern African Sub-region. Cambridge University Press, Cambridge. 2005.
34. Franco PM. Factors Affecting Hyrax Presence/Absence and Population Size: A case study in Serengeti National Park, Tanzania. Department of Biology. Norwey, Norwegian University of Science and Technology. 2015.
35. Kershenbaum A, Kershenbaum A, Blaustein L. Rock hyrax (*Procavia capensis*) den site selection: Preference for artificial sites. Wildl Res. 2011; 38: 244–248.
36. Wiid RE, Butler HJB. Population management of rock hyraxes (*Procavia capensis*) in residential areas. Pest Manag Sci. 2015; 71: 180–188.
37. Kagoro-Rugunda G. Crop raiding around Lake Mburo National Park, Uganda. Afr J Ecol., 2004; 42: 32–41.
38. Kifle ZA, Bekele A. Human–hamadryas baboon (*Papio hamadryas*) conflict in the Wonchit Valley, South Wollo, Ethiopia. Afr J Ecol. 2021; 59: 29–36.
39. Kifle Z. Human-olive baboon (*Papio anubis*) conflict in the human-modified landscape, Wollo, Ethiopia. Glob Ecol Conserv. 2021; 31: e01820.
40. Kifle Z, Bekele A. Human–gelada conflict and attitude of the local community toward the conservation of the southern gelada (*Theropithecus gelada obscurus*) around Borena Sayint National Park, Wollo, Ethiopia. Environ Manag. 2020; 65: 399–409.
41. Kifle Z, Assefa WW, Moges A. Human-hippo conflicts around Lake Tana Biosphere Reserve, Ethiopia: Vulnerability of hippopotamus in human-dominated landscape. PLoS ONE, 2023; 18: e0291802.
42. Power RJ. The Distribution and Status of Mammals in the North West Province. Department of Economic Development. Environment, Conservation and Tourism, North West Provincial Government, Mahikeng, South Africa. 2014.

List of tables

Table 1. Population size and structure of the rock hyrax during the dry and wet seasons.

Habitat type	Season							
	Dry				Wet			
	Adult	Juvenile	Total	%	Adult	Juvenile	Total	%
Human residence	137	51	188	44	162	70	232	45
Forest	26	2	28	7	31	12	43	8
Lakeshore	157	53	210	49	166	71	237	46
Bushland	-	-	-		-	-	-	
Total	320	106	426	-	359	153	512	-

Table 2. Socioeconomic and demographic profile of the respondents.

Characteristics	Frequency	Percentage
Sex		
Male	105	51.7
Female	98	48.3
Age		
21-35	29	14.3
Above 35	174	85.7
Educational level		
Illiterate	59	29.1
Primary school	43	21.2
high school	31	15.3
Certificate and above	18	8.8
Religion	52	25,6

Table 3: Attitude of respondents towards rock hyrax.

Educational level	Respondents‘ feeling		Total
	Good	Bad	
Primary (1-8)	21 (10.0%)	22 (11%)	43 (21.0%)
Secondary (9-12)	27 (14.0%)	3 (1.0)	30 (15.0%)
Certificate and above	19 (9.0%)	-	19 (9.0%)
Religion	16 (8.0%)	36 (18.0%)	52 (26.0%)
Illiterate	3 (1.0%)	56 (28.0%)	59 (29.0 %)

List of figure

Fig. 1. Map showing location of the study area in Ethiopia.

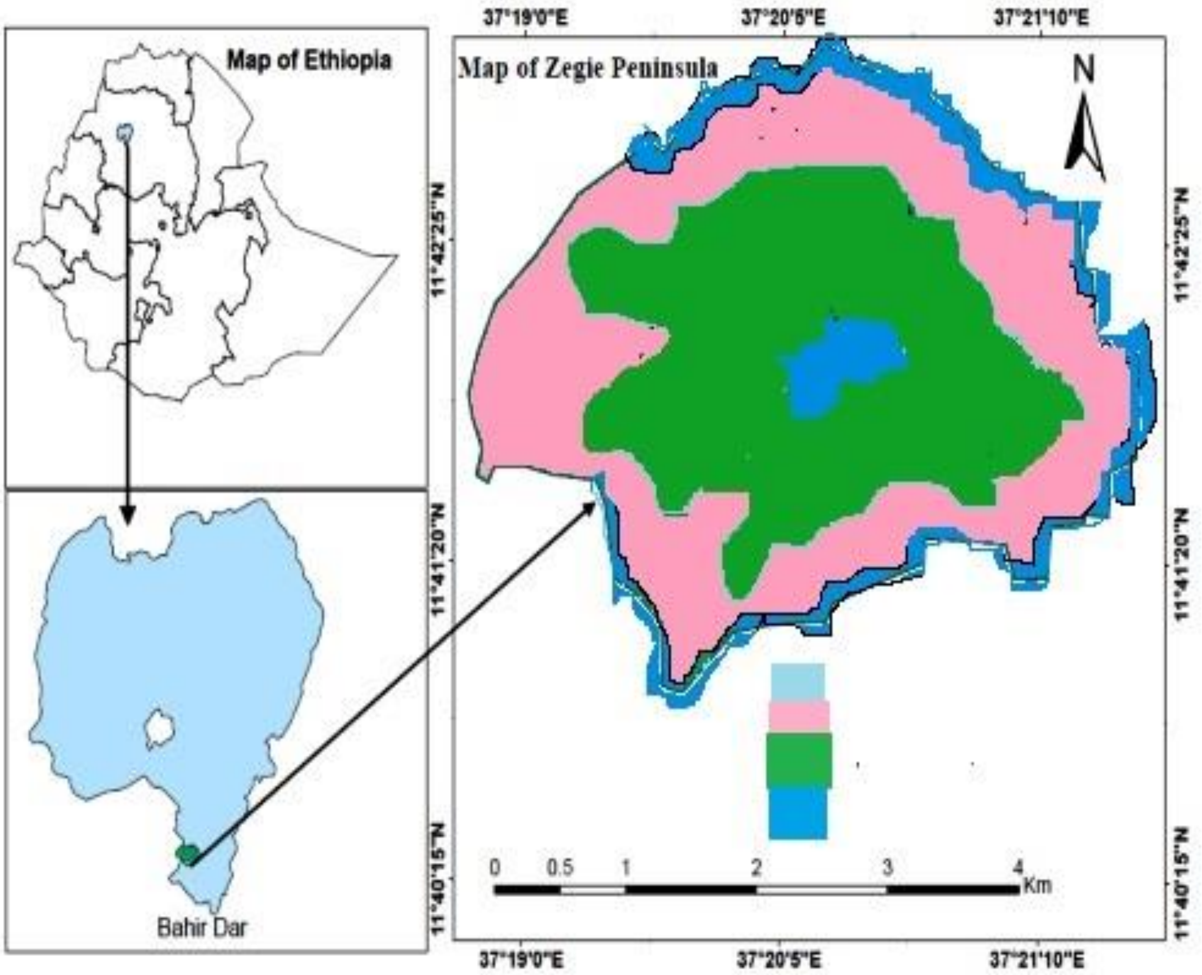

Fig. 2. Conservation attitude of rock hyrax in the region.

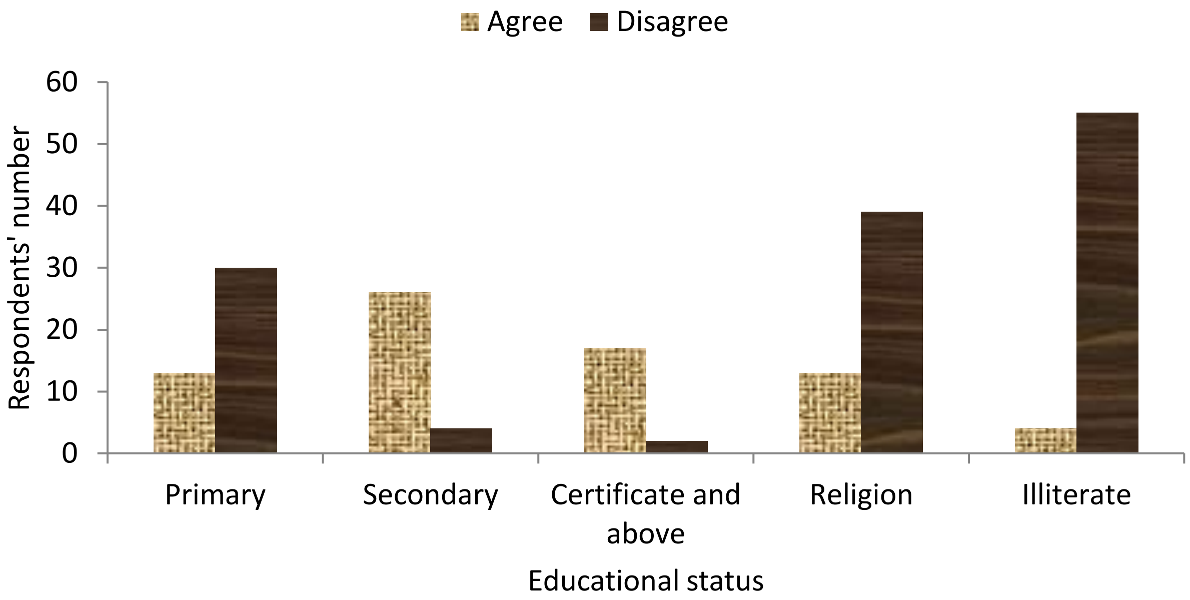

Supplement: Attachment — Submitted filename: PONE-D-24-32415 Marked.pdf [file pone.0323878.s003.pdf]
