# Supplementary material for: Population size, habitat association, and local residents’ attitude towards rock hyrax (Procavia capensis) in Zegie Peninsula, Ethiopia
Source: PLoS One. 2025 Jun 30;20(6):e0323878. doi: 10.1371/journal.pone.0323878 (PMC12208434; doi:10.1371/journal.pone.0323878)
Supplement: Attachment — Submitted filename: PONE-D-24-32415_reviewer Comments Embedded.pdf [file pone.0323878.s006.pdf]

PLOS ONE

Population size, habitat association and local residents’ attitude towards rock hyrax (Procavia capensis) in Zegie Peninsula, Ethiopia

--Manuscript Draft--

**Manuscript Number:**	PONE-D-24-32415R1
**Article Type:**	Research Article
**Full Title:**	Population size, habitat association and local residents’ attitude towards rock hyrax (Procavia capensis) in Zegie Peninsula, Ethiopia
**Short Title:**	Population size, habitat association and local residents’ attitude towards rock hyrax
**Corresponding Author:**	Zewdu Kifle, Ph.D<br>Bahir Dar University College of Science<br>Bahir Dar, ETHIOPIA
**Keywords:**	Conservation, population estimation, residents' attitude, rock hyrax, traditional medicine, Zegie Peninsula
**Abstract:**	Given the current rate of habitat loss particularly in the tropics, reliable data on the population size and habitats of wild animals is crucial to initiate conservation and management activities in an area. Wildlife ecologists have not studied the ecology of most medium-sized mammals in detail. Rock hyrax (Procavia capensis) is one of the medium-sized mammals that have not been well-studied in Ethiopia. Therefore, we conducted this study to determine the population size, and to examine the residents’ attitude towards the rock hyrax in the Zegie Peninsula, Ethiopia. We applied the strip transect counting method to estimate the population size and determine the density of rock hyrax in the area. We used structured questionnaires and informant interviews to examine the attitude of local people towards rock hyrax. We counted 469 individuals of rock hyraxes in the study area. Out of this population, adults comprised 72.4% and juveniles comprised 27.6%. Through extrapolation, we estimated the total population of c. 2,184 rock hyraxes within the Zegie Peninsula. The density of rock hyrax was 36.5 individuals/km2. The juvenile-to-adult age ratio was 1:2.6 in the area. Most rock hyraxes were found in the lakeshore habitat followed by human residence area. Most respondents had negative attitudes towards the rock hyraxes. Respondents reported that rock hyraxes caused pollution in the church and resident compounds due to their pellets and urine. They also claimed that rock hyraxes damaged fruits and vegetables. The respondents traditionally believed that rock hyraxes have a magical power that takes food from the local people. Most respondents reported that rock hyraxes’ body parts are used to prepare traditional medicines to cure human and cattle ailments. Key informants also stated that rock hyraxes’ body parts are used to treat cattle anthrax, and their pellets are used to treat leprosy. Most respondents did not support the conservation of rock hyraxes in the area. Our results provide a baseline for developing of conservation management strategies for the long-term survival of rock hyraxes.
**Order of Authors:**	Zewdu Kifle, Ph.D
	Birkie Alehegn, Msc
**Response to Reviewers:**	09 November 2024<br><br>Ethiopia<br>Ref.:<br>PONE-D-24-32415<br>Dear Editor in Chief<br>PLOS ONE<br>This is a revision of our manuscript entitled “Population size, habitat association and local residents’ attitude towards rock hyrax (Procavia capensis) in Zegie Peninsula, Ethiopia" by Birkie Alehegn and Zewdu Kifle. On 15 October 2024, we received a major revision request to be acceptable for publication. You invited us to resubmit the revised version of the manuscript that addressed the points raised during the review process.<br>Hence, we addressed all of the reviewers’ comments within this resubmitted manuscript.

Powered by Editorial Manager® and ProduXion Manager® from Aries Systems Corporation

Below there are lists of the revisions.
Sincerely,
Zewdu Kifle,
corresponding author
Response for Editor and reviewers
Thank you for your critical comments. I highlighted revisions with green color. In addition, I highlighted with red color and strikethrough those deleted letters, words, phrases, or sentences. In addition, I provided reviewers' concerns with justification within this file.
Thank you!
Response for Editor
Thank you! I have revised in detail on the presentation of this manuscript.
Response for reviewers
Reviewer #1:
Thank you for your important comments.
1.Population estimation methodology
Thank you! We determined the lengths and the numbers of transects in proportion to the size of the habitat strata. We repeated the same transects at each time of the day and season. We clarified why we have chosen those specific times of the day as the rock hyrax is mostly active for foraging and sun basking at these times of the day. We stated this in the revised version of the manuscript. We included confidence intervals.
2.Data on habitat association
Thank you! We noted why we did not record rock hyraxes in the bushland habitat. This habitat might not be suitable for foraging and hiding sites like rock outcrops. However, a habitat preference index or another statistical tool for the animal requires more habitat characteristics data. This parameter needs additional research to answer the question in more detail. Thus, we recommended to do further ecological research in the area
3.Questionnaire design and analysis
Thank you again! We performed a preliminary survey to ensure validity and reliability in the area. This pilot test was checked through data collection from 17 respondents. We stated the information in this revised version of the manuscript. We analyzed the data using the Chi-square test of independence to relate educational level and other factors to attitudes
4.Lack of ecological context and comparison
Food availability, better hiding places, and high human disturbances are considered factors for the low density of rock hyrax in the present study area compared to protected areas like the Bale Mountain National Park in Ethiopia and Serengeti National Park in Tanzania. We have stated such ecological aspects in the discussion section.
5.Cultural considerations
Thank you! It is very a fascinating cultural belief of the local community about the rock hyrax. That is why about 42.4% of the respondents had a good attitude towards rock hyrax in the study area. However, still more respondents had negative attitudes toward the animal. Yes, you are right. The traditional medicine value of the rock hyrax helps to promote conservation efforts in the region.
6.Conservation recommendations
Thank you! We added actionable recommendations to balance the needs of both the animals and the local people in the area
Reviewer #2
General comments:
Thank you for your critical comments on this research work.
1.Thank you for appreciating the usefulness of the study.
2. Thank you! We corrected all reference citations.
3.Thank you we deleted this local name of rock hyrax 'Shembeko'
4.Thank you here again! To avoid double counting, we counted consecutive transects simultaneously. The distance between two consecutive transect lines was 200 to 300 m to avoid double counting. Therefore the likelihood of double counting is almost zero. We scanned the habitat of rock hyrax within the strip with brief pauses of two minutes every 50 m. We stated this in the revised version of the manuscript.
5.Thank you! Sorry, we used the Ethiopian time zone. We corrected it.
6.We put the overall mean number of rock hyraxes of each habitat stratum.
7.Thank you! We added the reason why we counted a high proportion (44/45%) of rock hyraxes in the human dwellings in the discussion section.

Powered by Editorial Manager® and ProduXion Manager® from Aries Systems Corporation

	8.In tables 2 &3 religion, these are deacons and priests who learned religion through the Ethiopian Orthodox Church. We designed respondents who completed grade 12 as certificates and above.<br><br>Reviewer #3:<br>Thank you for appreciating the manuscript's information. I corrected concerns that you raised in the annotated pdf.<br><br>Specific comments:<br><br>1. Line 11: Thank you! We changed the word 'well' with 'in detail'.<br>2.Line 27 and 33. We changed the word 'region' into area<br>3.Line 101 and 102: Thank toy! We rewrote the sentences.<br>4.Line 16: We deleted 'Shenbeko'.<br>5.Line 168: We deleted 'a total mean of' and we changed 'Zegie Peninsula' with study 'area'.<br>6.Line 191: Thank you! We added 'a' before 'few'.<br>7.Line 195-201: We edited based on your comment.<br>8.Line 204-205: We corrected<br>9.Line 219: We deleted 'some' from the sentence.<br>10.Line 229-220: We edited the sentence.<br>11.Line 247: Thank you! We corrected the sentence based on your comments.<br>12.Line 246: We edited the phrase 'habitat disturbance'.<br>13.Line 251-254: we corrected those sentences. We put the reference '13' as the end of the sentence.<br>14.Lien 256: We put the reference at the end of the sentence.<br>15.Line 259: We corrected it.<br>16.Line 262: We deleted 'in the region'.<br>17.Line 267: We edited it.<br>18.Line 269 and 270: We put these references at the end of the sentence.<br>19.Line 280: We deleted 'save'<br>20.Line 282, 284, 285, and 287: We put the reference at the end of the sentence.<br>21.Line 286: We deleted letter 'y' from rocky.<br>22.Line 292: We edited this sentence.<br>23.Line 295: Thank you! We edited this sentence.<br>24.Line 297 and 298: We edited these sentences.<br>25.Line 298: We put the reference at the end of the sentence.<br>26.Line 300-301: We deleted this sentence.<br>27.Line 303: We deleted the word 'background'.<br>28.Line 309: We deleted the word 'enough'.<br>29.Line 310: We put the reference at the end of the sentence.<br>30.Line 312: We corrected this sentence.<br>31.Line 317: Thank you! We corrected this sentence.<br>32.Line 332: We corrected this sentence.
**Additional Information:**	
**Question**	**Response**
**Financial Disclosure**<br><br>Enter a financial disclosure statement that describes the sources of funding for the work included in this submission. Review the submission guidelines for detailed requirements. View published research articles from *PLOS ONE* for specific examples.<br><br>This statement is required for submission and **will appear in the published article** if	Yes

the submission is accepted. Please make sure it is accurate.<br><br>**Funded studies**<br>Enter a statement with the following details:<br>• Initials of the authors who received each award<br>• Grant numbers awarded to each author<br>• The full name of each funder<br>• URL of each funder website<br>• Did the sponsors or funders play any role in the study design, data collection and analysis, decision to publish, or preparation of the manuscript?<br><br>Did you receive funding for this work?	
Please add funding details.<br>as follow-up to "**Financial Disclosure**<br><br>Enter a financial disclosure statement that describes the sources of funding for the work included in this submission. Review the submission guidelines for detailed requirements. View published research articles from *PLOS ONE* for specific examples.<br><br>This statement is required for submission and **will appear in the published article** if the submission is accepted. Please make sure it is accurate.<br><br>**Funded studies**<br>Enter a statement with the following details:<br>• Initials of the authors who received each award<br>• Grant numbers awarded to each author<br>• The full name of each funder<br>• URL of each funder website<br>• Did the sponsors or funders play any role in the study design, data collection and analysis, decision to publish, or preparation of the manuscript?<br><br>Did you receive funding for this work?"	This work was funded by the Bahir Dar University The funders had no role in study design, data collection and analysis, decision to publish, or preparation of the manuscript.
Please select the country of your main	ETHIOPIA - ET

research funder (please select carefully as in some cases this is used in fee calculation).<br>as follow-up to "**Financial Disclosure**<br><br>Enter a financial disclosure statement that describes the sources of funding for the work included in this submission. Review the submission guidelines for detailed requirements. View published research articles from *PLOS ONE* for specific examples.<br><br>This statement is required for submission and **will appear in the published article** if the submission is accepted. Please make sure it is accurate.<br><br>**Funded studies**<br>Enter a statement with the following details:<br>• Initials of the authors who received each award<br>• Grant numbers awarded to each author<br>• The full name of each funder<br>• URL of each funder website<br>• Did the sponsors or funders play any role in the study design, data collection and analysis, decision to publish, or preparation of the manuscript?<br><br>Did you receive funding for this work?"	
**Competing Interests**<br><br>Use the instructions below to enter a competing interest statement for this submission. On behalf of all authors, disclose any competing interests that could be perceived to bias this work—acknowledging all financial support and any other relevant financial or non-financial competing interests.<br><br>This statement is required for submission and **will appear in the published article** if the submission is accepted. Please make sure it is accurate and that any funding sources listed in your Funding Information	The authors have declared that no competing interests exist.

later in the submission form are also declared in your Financial Disclosure statement.<br><br>View published research articles from *PLOS ONE* for specific examples.<br><br>**NO authors have competing interests**<br><br>Enter: *The authors have declared that no competing interests exist.*<br><br>**Authors with competing interests**<br><br>Enter competing interest details beginning with this statement:<br><br>*I have read the journal's policy and the authors of this manuscript have the following competing interests: [insert competing interests here]*<br><br>* typeset	
**Ethics Statement**<br><br>Enter an ethics statement for this submission. This statement is required if the study involved:<br><br>• Human participants<br>• Human specimens or tissue<br>• Vertebrate animals or cephalopods<br>• Vertebrate embryos or tissues<br>• Field research<br><br>Write "N/A" if the submission does not require an ethics statement.<br><br>General guidance is provided below. Consult the submission guidelines for detailed instructions. **Make sure that all information entered here is included in the Methods section of the manuscript.**	This study was carried out according to the guidelines of the review board of Bahir Dar University (BDU) of the human ethics research. The interviewer fully informed to the respondents on the aim of the research and how the data obtained from them would be used. Confidentiality of information was assured by the investigator. Participants gave their consent verbally after being explained the aim of the survey and how the interview would proceed. Consent was obtained by having participants state that they agree to participate.

**Format for specific study types**<br><br>**Human Subject Research (involving human participants and/or tissue)**<br>• Give the name of the institutional review board or ethics committee that approved the study<br>• Include the approval number and/or a statement indicating approval of this research<br>• Indicate the form of consent obtained (written/oral) or the reason that consent was not obtained (e.g. the data were analyzed anonymously)<br><br>**Animal Research (involving vertebrate animals, embryos or tissues)**<br>• Provide the name of the Institutional Animal Care and Use Committee (IACUC) or other relevant ethics board that reviewed the study protocol, and indicate whether they approved this research or granted a formal waiver of ethical approval<br>• Include an approval number if one was obtained<br>• If the study involved *non-human primates*, add *additional details* about animal welfare and steps taken to ameliorate suffering<br>• If anesthesia, euthanasia, or any kind of animal sacrifice is part of the study, include briefly which substances and/or methods were applied<br><br>**Field Research**<br><br>Include the following details if this study involves the collection of plant, animal, or other materials from a natural setting:<br>• Field permit number<br>• Name of the institution or relevant body that granted permission	
**Data Availability**<br><br>Authors are required to make all data underlying the findings described fully available, without restriction, and from the time of publication. PLOS allows rare exceptions to address legal and ethical concerns. See the PLOS Data Policy and FAQ for detailed information.	Yes - all data are fully available without restriction

A Data Availability Statement describing where the data can be found is required at submission. Your answers to this question constitute the Data Availability Statement and **will be published in the article**, if accepted.<br><br>**Important:** Stating 'data available on request from the author' is not sufficient. If your data are only available upon request, select 'No' for the first question and explain your exceptional situation in the text box.<br><br>Do the authors confirm that all data underlying the findings described in their manuscript are fully available without restriction?	
**Describe where the data may be found in full sentences. If you are copying our sample text, replace any instances of XXX with the appropriate details.**<br><br>• If the data are **held or will be held in a public repository**, include URLs, accession numbers or DOIs. If this information will only be available after acceptance, indicate this by ticking the box below. For example: *All XXX files are available from the XXX database (accession number(s) XXX, XXX.*).<br>• If the data are all contained **within the manuscript and/or Supporting Information files**, enter the following: *All relevant data are within the manuscript and its Supporting Information files.*<br>• If neither of these applies but you are able to provide **details of access elsewhere**, with or without limitations, please do so. For example:<br><br>*Data cannot be shared publicly because of [XXX]. Data are available from the XXX Institutional Data Access / Ethics Committee (contact via XXX) for researchers who meet the criteria for access to confidential data.*<br><br>*The data underlying the results presented in the study are available from (include the name of the third party*	All relevant data are within the manuscript. We can share the data when requested based on the PLOS ONE guidelines.

and contact information or URL). • This text is appropriate if the data are owned by a third party and authors do not have permission to share the data. <br>* typeset	
Additional data availability information:	Tick here if the URLs/accession numbers/DOIs will be available only after acceptance of the manuscript for publication so that we can ensure their inclusion before publication.

Powered by Editorial Manager® and ProduXion Manager® from Aries Systems Corporation

Manuscript

Click here to access/download;Manuscript;Zewdu Kifle-Population size of rock hyrax-Unmarked version.docx

Population size, habitat association and local residents' attitude towards rock hyrax (*Procavia capensis*) in Zegie Peninsula, Ethiopia

Birkie Alehegn[1], Zewdu Kifle*[1]

[1]Department of Biology, Bahir Dar University, Bahir Dar, Ethiopia

Corresponding author: Zewdu Kifle, email: zewdu96@yahoo.com;

ORCID: https://orcid.org/0000-0002-0014-3525

Abstract

Given the current rate of habitat loss particularly in the tropics, reliable data on the population size and habitats of wild animals is crucial to initiate conservation and management activities in an area. Wildlife ecologists have not studied the ecology of most medium-sized mammals in detail. Rock hyrax (*Procavia capensis*) is one of the medium-sized mammals that have not been well-studied in Ethiopia. Therefore, we conducted this study to determine the population size, and to examine the residents' attitude towards the rock hyrax in the Zegie Peninsula, Ethiopia. We applied the strip transect counting method to estimate the population size and determine the density of rock hyrax in the area. We used structured questionnaires and informant interviews to examine the attitude of local people towards rock hyrax. We counted 469 individuals of rock hyraxes in the study area. Out of this population, adults comprised 72.4% and juveniles comprised 27.6%. Through extrapolation, we estimated the total population of c. 2,184 rock hyraxes within the Zegie Peninsula. The density of rock hyrax was 36.5 individuals/km^2. The juvenile-to-adult age ratio was 1:2.6 in the area. Most rock hyraxes were found in the lakeshore habitat followed by human residence area. Most respondents had negative attitudes towards the rock hyraxes. Respondents reported that rock hyraxes caused pollution in the church and resident compounds due to their pellets and urine. They also claimed that rock hyraxes damaged fruits and vegetables. The respondents traditionally believed that rock hyraxes have a magical power that takes food from the local people. Most respondents reported that rock hyraxes' body parts are used to prepare traditional medicines to cure human and cattle ailments. Key informants also stated that rock hyraxes' body parts are used to treat cattle anthrax, and their pellets are used to treat leprosy. Most respondents did not support the conservation of rock hyraxes in the area. Our

results provide a baseline for developing of conservation management strategies for the long-term survival of rock hyraxes.

Keywords: Conservation, population estimation, residents' attitude, rock hyrax, traditional medicine, Zegie Peninsula

Introduction

The biological diversity around the globe represents a resource that benefits the current and future generations [1]. However, because of the need to feed a growing human population, forests have been cut down for agriculture, livestock grazing, settlement, and urbanization [2, 3]. This has led to the fragmentation of the landscape and has altered the distribution and abundance of wild animals and plants [4]. The effect and magnitude of fragmentation on biodiversity is complex [4, 5]. The human population in Africa is growing at a rapid rate which needs space to live and harvest. Human interference in the form of clearing animal habitats for agriculture has impacted African wildlife [6].

Ethiopia possesses different altitude ranges and topography [7]. The altitudinal variation of the country ranges from 126 meters below sea level in the Danakil Depression of the Afar region to the highest peak of 4,620 meters above sea level on Mount Ras Dejen of the Simien Mountains National Park. These variations in altitude and topography create diverse climatic conditions varying from the hot and dry desert in the lowland areas to cold and humid alpine habitats in the highlands. These factors strongly influence Ethiopia's extraordinary range of terrestrial and aquatic ecosystems which have contributed to high flora and fauna diversity and rate of endemism [8, 9].

Hyraxes are medium-sized terrestrial mammals native to Africa and the Middle East. Hyrax comprises three extant genera: *Procavia*, *Heterohyrax,* and *Dendrohyrax*. Five extant species of hyraxes are recognized: the rock hyrax (*Procavia capensis*) and the yellow-spotted rock hyrax (*Heterohyrax brucei*), which both live on rock outcrops, including cliffs in Ethiopia and isolated granite outcrops called koppies in southern Africa; the western tree hyrax (*Dendrohyrax dorsalis*), southern tree hyrax (*D. arboreus*), and eastern tree hyrax (*D. validus*). Their distribution is limited to Africa, except for *P. capensis* which is also found in the Middle East.

Rock hyraxes (*P. capensis*) look like a large guinea pig or a rabbit with rounded, short ears and no visible tail. The rock hyrax's coat color ranges from yellowish to grayish-brown, with a

lighter underbelly. They have short legs very long bodies that are concealed by a hunched posture and long fur [10]. They have long, tactile hairs, especially on the muzzle, throat, cheeks, and rump. They eat mostly grass, fruits, and leaves [10, 11]. They have disproportionately large jaws and the ability to draw the lips right back allowing them to bite off and chew large mouthfuls of vegetation at a time. Their habitats include the savanna, shrubland, desert, and rocky areas, such as cliffs and mountain peaks [10]. Rock hyraxes are slow-moving with only a few defensive mechanisms against predators. They depend on rock outcrops, which provide protection from predators, access to safe foraging areas, and good vantage points [12].

Rock hyraxes (*P. capensis*) live in rock outcrops, piles of boulders, and fractured cliff faces [13]. They have the widest geographical and altitudinal distribution among the hyraxes [14]. Even if there are no major threats to rock hyraxes, predation, intra-specific competition, immigration, territorial fighting, and dispersal regulate their population. They are hunted by people for their meat and soft fur. For example, the Hadza or Watindiga, a bushman tribe in Tanzania, hunt rock hyraxes for food [15]. In some areas of Egypt, they are hunted for food [16]. In countries like Kenya and Israel, rock hyraxes are not allowed to live in their localities for being reservoirs of cutaneous *leishmaniasis* [17].

Rock hyrax is classified as a species of Least Concern Red List of Threatened Species [18] However; their populations have declined due to habitat loss, disease, and predation [19]. They are considered a pest in some areas, competing with farmers by eating their crops. They are also subjected to threats from various avian and mammalian predators.

Assessing the population size of wild animals and their habitat association as well as understanding the attitudes of the local people towards the wild animal is important for designing sustainable conservation and management strategies [20]. Only a few studies have been conducted on the population size, distribution, and ecology of rock hyrax in Ethiopia [21, 22, 23]. Therefore, the population size and distribution of rock hyrax are poorly known and documented in the country. Specifically, there is no information on the population size of rock hyrax in the Zegie Peninsula, Ethiopia. In addition, the attitude of local people towards rock hyrax has not studied in Ethiopia. Therefore, the objectives of the study were (1) to determine the population size of rock hyrax in the Zegie Peninsula**;** (2) to obtain information on the habitat association of rock hyrax in the area; and (3) to examine the attitude of local people towards rock

hyrax conservation. Our findings provide baseline information for further studies (e.g., traditional medicine of rock hyrax and other ecological and behavioral aspects) in the area.

Materials and Methods

Ethical considerations

This study was carried out according to the guidelines of the review board of Bahir Dar University (BDU) of human ethics research. The interviewer fully informed the respondents about the aim of the research and how the data obtained from them would be used. Confidentiality of information was assured by the investigator. Participants gave their consent verbally to participate and agreed after being explained the aim of the survey and how the interview would proceed.

Study area

We carried out this study in the Zegie Peninsula, the southwestern shore of Lake Tana, Ethiopia. Zegie Peninsula covers 13.47 Km^2. The Zegie Peninsula is situated at 11°40’ to 11°43’ N latitude and 37°19’ to 37°21’ E. It is found at elevations between 1775 m and 1985 m above sea level. Zegie Peninsula is home to many Ethiopian Orthodox churches and monasteries dating back to the 13th century with a unique cultural, and religious heritage, and historical and aesthetic value. It is an important site for the persevering religion and culture of the country. It is one of the tourist destination sites in Ethiopia. The climate of Zegie Peninsula is a warm-temperate tropical highland monsoon with a mean temperature of 21.9°C. The area experiences two main seasons: the wet season and the dry season. The wet season typically occurs between June and October, while the dry season occurs between November and May.

The Peninsula is surrounded by Lake Tana in the northern and eastern directions, wetlands in its western parts, and farmlands in the southern directions. The dominant vegetation of the area are *Mimusops kumme, Syzygium guineense, Ficus vasta*, *Jasminum grandiflorum*, *Momordica foetida*, *Clematis simensis*, *Tragia brevipes, Urera hypselodendron and Phytolacca dodecandra.* The Zegie Peninsula is also home to different mammalian species like leopard (*Panthera pardus*), grivet monkey (*Chlorocebus aethiops*), rock hyrax (*Procavia capensis*), bushpig (*Potamochoerus larvatus*), and duiker (*Sylvicapra grimmia*). In addition, the peninsula supports diverse species of birds, amphibians, reptiles, mollusks, and insects. However, its wild

animals and their habitats are under threat due to deforestation and illegal hunting practices [24]. For example, common bushbuck (*Tragelaphus scriptus*) and warthog (*Phacochoerus africanus*) have been eradicated due to illegal hunting in the area [25].

Population size and habitat association of rock hyraxes

Between January 10, 2021 and June 15, 2021, we applied a line transect method to estimate the population size of rock hyrax in the study area. The census zone was divided into several sample units. The selection of the sampling units was done by using two-stage sampling techniques. We divided the study area into four habitat strata: The lakeshore stratum of the coffee-dominated semi-natural forest, human premises, tall trees with coffee plantations, and bushland. We established 18 transect lines in these four habitats. The average lengths of the line transects were 4.12 km in the human residence, 4.42 km in the forest, 1.83 km in the bushland, and 0.69 km in the forest. The distance between two consecutive transect lines was 200 to 300 m to avoid double counting. Therefore, the possibility (likelihood) of double counting is almost zero using this distance gap between consecutive transects for the target species. The length and the number of transects were proportional to the size of each habitat stratum. During the transect survey, we recorded the number of rock hyrax seen, the age category, and the habitat type. We conducted transect walks for three consecutive days per month in the morning (9:00–11:30 a.m.) and in the late afternoon (8:30–11:00 p.m.) when the rock hyrax is active for foraging and sun basking at these day times. We surveyed transects by direct counting method walking at an average speed of 1km/hr [26]. Within the line transect, we moved along the lines, looking for the target species with a 25 m fixed transect width to the left and right sides of the line [27]. We paused two minutes every 50 m to allow for the scanning of vegetation and rocky outcrops within the strip for the presence of the resting rock hyraxes. We sampled 2.71 km^2 from the total size of 12.62 km^2 area. The study covered the dry (February, March, and April) and wet (June, July, and September) seasons. We repeated the same transect during the dry and wet seasons and at different times of the day.

Questionnaire survey

Between March 16, 2021 and June 20, 2021, we used a structured questionnaire interview method to collect data on the attitude of local people towards rock hyrax. The questionnaire was

pre-tested on a sample of 17 respondents to ensure its validity and reliability and to improve its clarity in the study area. We applied a two-stage sampling technique to select the respondents. First, we selected seven villages from two *kebeles* (i.e., Ura and Yiganda-Mehale Zegie) using purposive sampling. Second, we selected the household heads (respondents) from the sampling villages by systematic random sampling method using the recorded book found in the *kebele* (local office). We interviewed 210 respondents out of 1,116 households inhabiting the Peninsula, yielding a sampling intensity of 18.82%. However, we discarded seven questionnaires since they were not fully answered by the respondents. We designed the structured questionnaire to examine the attitudes, practices, and beliefs of the local people, knowledge of its significance on rock hyrax as well as to identify factors that influence community perceptions on rock hyraxes. The questionnaire also included respondents' level of education, age, sex, and livelihood. To triangulate the data collected by questionnaire, we undertook key informant interviews with nine dwellers (community leaders, elders, religious leaders, and association leaders) to express the culture and practice related to rock hyrax in the study area.

Data analysis

We summed up all transects of each habitat results to determine the total population of rock hyraxes [28]. We extrapolated the transect result to estimate the total population number of rock hyrax within the study area. We excluded bushland habitat from the analysis since we did not record any rock hyrax in this habitat during the study period. We determined the density of the rock hyrax population by dividing the counted population (individuals) in the sample sites by the total area of the study area (km^2). We analyzed data using descriptive statistics, frequencies, and percentages. We used the Chi-square test of independence to test the hypothesis that categorical variables (gender vs. attitude towards rock hyrax as well as education level vs. attitude towards rock hyrax) are independent of each other. We set the level of rejection for all tests at $\alpha = 0.05$.

Results

Population size of rock hyrax

We counted 469 individuals of rock hyrax in the study area. Out of this population, the number of adults was 339.5 (72.4%) and juveniles 129.5 (27.6%). Through extrapolation, the total estimated population size of rock hyrax was 2184 individuals within the upper limit of 2648 and

lower limit of 1720 with a 95% confidence interval of the difference in the Zegie Peninsula. The density of rock hyrax was 37.2 individuals/km^2 in the Peninsula. The adult-to-juvenile age ratio was 1:2.6.

From the total population, we counted 426 individuals of rock hyrax during the dry season and 512 during the wet season (Table 1). During the dry season, adults constituted 75.1%, and babies (juveniles) 24.9 %. While, during the wet season adults constituted 70.1% and babies 29.9%. Adult to juvenile ratio was 1:3.0 during the dry season and 1:2.3 during the wet season. The density of rock hyrax during the dry season was 33 individuals/km^2 and 40 individuals/km^2 during the wet season.

We recorded the highest numbers of rock hyrax in the lakeshore (223.5, 47.7%) and the lowest in the forest habitat (35.5, 7.5%). We recorded 210 (44.8%) individuals in the human residence area (Table 1). We didn't record rock hyrax in the bushland habitat. We recorded the highest population size of rock hyraxes in the lakeshore habitat during the dry (210 individuals) and wet (237 individuals) seasons.

Questionnaire survey

Socio-demographic features of the respondents

We conducted the questionnaire interviews with 203 respondents of which 105 were males and 98 were females with ages ranging from 21 to 65 (Table 2). Most (97.5%) respondents reported that their livelihood depends on a coffee plantation, fruit production, fishing, wood (log) selling for firewood, small-scale trading, and handcraft making and selling. Most (97.0%) respondents had their farmlands. Most (71.4%) respondents did not have sheep while other (28.6%) respondents had sheep. A few (2.5%) respondents were governmental and non-governmental employees. Many (29.1%) respondents had not attended both formal and informal education.

Local residents' attitudes towards rock hyraxes

Over half (57.6%) of respondents had negative attitudes towards rock hyraxes. On the contrary, other (42.4%) respondents had positive attitudes towards rock hyraxes (Table 3). These positive feelings respondents expressed that they used rock hyrax as a food source for their domestic dogs and income by selling its hide and meat to traditional healers. Male respondents had better positive feelings about the presence of rock hyraxes in the area than female respondents, and

there was a statistically significant relationship between gender and attitudes towards rock hyraxes ($\chi2$ = 27.37, df = 1, *P* = 0.001). Higher levels of educational background respondents had better positive feelings about the presence of rock hyraxes. The proportion of positive attitudes towards rock hyraxes among the respondent's category of educational background was statistically significantly different ($\chi2$ = 90.91, df = 4, *P* = 0.001).

Most (64.5%) respondents disagreed with the idea of rock hyrax conservation in the area while other (35.5%) respondents agreed with it. Most uneducated, religious, and primary school level respondents disagreed on rock hyraxes' protection; however, many secondary school and higher educational level respondents agreed on the conservation of rock hyraxes in their localities (Fig. 1). There was a statistically significant relationship between the level of education and conservation attitude towards rock hyraxes ($\chi2$ = 82.26, df = 4, P = 0.001).

Perceived costs and benefits of rock hyrax presence

Most (80.0%) respondents reported that rock hyrax damaged fruits and vegetables. While a few (20%) respondents believed that rock hyrax did not cause significant crop damage in their localities. Similarly, the key informant interview participants deeply expressed the destructive behavior of rock hyrax on crops. In addition, many respondents reported that economically important plants like *Rhamnus prinoides*, *Carica papaya*, *Citrus lemon*, *Eucalyptus camaldulesis*, *Mimusops kummel*, and *Acacia pilispina* are damaged by rock hyraxes. Similarly, key informants also deeply expressed the destructive behavior of rock hyrax on plants.

Many local people had bad traditional myths about the rock hyraxes which they have a magic power (locally known as "*wosaji*"). They claimed that rock hyraxes take food magically when they see people eating. In addition, the other common problem claimed by key informants was the occurrence of rock hyrax pellets and urine that created pollution (bad smell) in and around churches and resident compounds.

Most (93.0%) respondents replied that rock hyraxes have benefits. Of these respondents, 88.0% described that rock hyrax body parts are used to prepare traditional medicines to cure human and cattle ailments. While, a few (3.0%) respondents reported that the skin of rock hyrax is used as medicine (specific diseases were not mentioned), and a few (2.0%) respondents used rock hyraxes for their dog's food. Key informants also reported the medicinal value of rock hyrax in which its flesh is used to treat cattle anthrax and the pellets are used to treat leprosy.

Awareness of rock hyrax population and conservation status

When we asked about the population size of rock hyraxes, 112 (55.0%) respondents reported that the population size has decreased in the area, 76 (37.0%) believed the number increased in recent years, and 15 (8.0%) had no information whether they increased or decreased. Many (56.0%) respondents have sawed most frequently three individuals of rock hyraxes in a group while other (44.0%) respondents have sawed four to five individuals in a group. Most key informants also informed that rock hyraxes were not seen as widely in lakeshores and church compounds as they were seen years before. They reported that hunting for traditional medicine and dogs and habitat disturbances like cutting trees and stone fencing are responsible for their decrement in the area.

Controlling mechanisms

Many (57.0%) respondents used killing and other (43.0%) respondents used chasing as a means of controlling crop damage caused by rock hyrax and avoiding pollution by its pellets and urine. Local people killed rock hyrax with spears, poisons, traps, and dogs. They chased away rock hyrax by throwing stones, with dogs, and making noise.

Conservation threats to rock hyraxes

In the Zegie Peninsula, 115 (56.0%) respondents reported threats to rock hyraxes in their locality, while other (44.0%) respondents stated that there is no threat to rock hyraxes. Of the 115 respondents who reported threats to rock hyraxes, most (65.2%) respondents considered hunting and killing to be the main threat followed by predation by dogs (28.8%) and 6.0% human-based habitat disturbances.

Discussion

Most researchers do not study the population and behavioral ecology of medium-sized mammals and their conflicts with local people. In the current study, we investigated the population size of rock hyrax and the extent of human-rock hyrax conflict in the Zegie Peninsula, Ethiopia. We found 469 individuals of rock hyraxes in the Peninsula. Adults constituted more than babies (juveniles). The number of more adults than babies is also recorded in the Bale Mountains National Park, Ethiopia [13]. This might be due to predation pressure on young individuals than adults. In addition, on many occasions babies might hide in their shelter causing them difficulty to be seen by the observers during transect walks.

In the present study, the average density of rock hyrax was 37.2 individuals/km^2. On the contrary, the mean population density of 664.5 and 529.9 individuals/km^2 during the wet and dry seasons was reported in the Bale Mountain National Park [13]. Rock hyrax population density studies elsewhere have also revealed a density of 20 to 100 individuals/ km^2 in Mount Kenya [29], 500 to 4,000 individuals/km^2 in the Serengeti National Park, Tanzania [19], and 73 to 94 individuals/km^2 in the Matobo National Park, Zimbabwe [28]. The minimum availability of rock outcrops, cliffs with crevices, caves, and gorges having rock piles or crevices in the Zegie Peninsula might be the case for low population density. In addition, high habitat disturbance in the current unprotected study area might be the cause for the minimum density of rock hyrax. Severe habitat alterations due to various human activities in the natural habitat influence the ecology and population dynamics of wild animals [30]. Natural habitat disturbance through human activities influences the population size and density of rock hyrax. The highest population size was recorded in areas where rock outcrops, cliffs, grasses, and shrubs are available [13]. It is also expected that protected areas like the Bale Mountains National Park, Ethiopia, and the Serengeti National Park, Tanzania, accommodate a higher density of wild animals than a human-modified landscape like the current study area.

An extreme population fluctuation of hyrax is recorded at Montopos in Zimbabwe [11] and in the Serengeti National Park of Tanzania. [19]. In Augrabies Falls National Park, South Africa, it is also found that hyrax populations fluctuate mainly due to predation, food availability, drought, and infectious disease [31]. In the present study, we counted more rock hyrax populations during the wet season than in the dry season. This might be the birth of more babies during the wet season when the availability of food that can satisfy their nutritional demands is higher. The wet season is full of nutritious food items, which could promote the breeding of animals and birth. Hyrax population fluctuation between seasons is mainly due to predation and food availability [31, 32]. During the dry season, hyraxes expose themselves to greater predation risk as they seek food farther from their covers and refuges [13, 31].

Rock hyraxes were not uniformly distributed in the study area. For example, we did not record rock hyrax in the bushland since this habitat might not be suitable for foraging and hiding sites like rock outcrops and boulder piles. However, we counted more rock hyraxes in the lakeshore habitat. This might be due to the availability of abundant rock outcrops for rock hyraxes in this habitat to forage and shelter. Similarly, rock hyrax is not uniformly distributed in

the Bale Mountains National Park, Ethiopia [13]. The distribution of rock hyrax mainly depends on the availability of food and shelter that allows it to hide and watch its predators easily. The distribution of rock hyraxes is delineated by the presence of appropriate shelter [29]. Likewise, rock hyraxes are typically associated with rock outcrops, cliffs, or piles of boulders with bushes [33]. The distribution of rock hyrax also decreases around human premises due to habitat loss and human disturbance [34]. However, a significant number of rock hyraxes also dwelled around human dwellings in the current study area. This might be the availability of hiding places since local people make compound fences using rocks. These rock fences might help rock hyraxes from being attacked by potential predators. In addition, as animals have started to dwell in human-modified landscapes, they adapt to human disturbances in the area by using the available resources. This ecological and behavioral adaptation of rock hyraxes might help to roam and forage around human dwellings. However, further assessment of habitat characteristics is needed to confirm our justification in the present study area.

Most people did not like the presence of rock hyraxes in their locality. This might be due to the destructive behavior of fruits and vegetables. In addition, the traditional belief of the local people on the rock hyrax's magic power to take their food might develop negative perceptions toward the animal. Rock hyraxes are reservoirs of cutaneous *leishmaniasis* that can lead to disease outbreaks around human settlements [17, 35]. Members of rock hyrax colonies urinate and defecate in designated areas that might cause bad smells in the church and residence compounds. These behaviors of rock hyrax might be the reasons that local people develop a negative attitude toward this animal. On the other hand, even though traditional medicine helps to promote conservation efforts, the local communities are bothered by the effects on the animals.

In the present study, respondents reported that rock hyrax damages crops. When rock hyraxes inhabit rock boulders or cliffs near farmlands they damage crop seedlings [13. 21]. It is also documented that rock hyraxes inhabiting close to farmlands damage crops [36]. Similar studies in different parts of Africa also revealed that wild animals posed main threats to crops and livestock [37, 38, 39].

A strong attitudinal difference was seen towards rock hyrax presence related to the educational level of respondents. Respondents of higher educational backgrounds had better feelings about rock hyrax presence in their locality than the uneducated and religious informal

educational backgrounds. Moreover, males had a better positive feeling in the presence of rock hyraxes in their locality than females. Crop damage and livestock predation by wild animals influence the attitude of local farmers toward their conservation [38, 39, 40, 41].

Most uneducated and primary school level respondents disagreed with the idea of rock hyrax conservation. This indicated that most of the dwellers did not have awareness and had less concern for rock hyrax protection. It is also noted that there have been concerns about the species by the landowners in the Magaliesberg province, North West Region of South Africa [42]. On the contrary, most of the secondary school and above educational level respondents agreed on the protection of rock hyraxes in the area. Therefore, formal education is crucial to developing wildlife conservation initiatives in an area.

Conclusions

This study provides crucial information on the population size and habitat association of rock hyraxes as well as the local peoples' attitude towards rock hyraxes and their ethnozoological importance in inhabiting the Zegie Peninsula, Ethiopia. We identified the Peninsula as an important site for the conservation of rock hyrax. However, human disturbances, mainly the absence of enough shelter, affect the population density of rock hyrax in the area.

Even if rock hyraxes damaged crops, local people use rock hyraxes as a traditional medicine to treat human and livestock aliments. This practice is important to promote conservation measures for rock hyrax in Ethiopia. The local administration and other governmental and non-governmental organizations should work cooperatively to create awareness and educate the local community about the economic and ecological importance and conservation of rock hyrax. We recommend that local people grow cash crops like coffee, mango, and avocado in the area to reduce conflicts between the local people and rock hyraxes regarding crop damage. Local people should create alternative job opportunities like fish pond farming and livestock fattening to benefit economically. In addition, it is crucial to promote the medicinal values of rock hyraxes among the local community and other stakeholders. These actions help as the habitat management strategies to minimize the rock hyrax habitat from further alternations and destructions. These strategies also help to balance the needs of both the animals and the people. This study provides baseline information for further studies on the medicinal values of rock hyrax as well as ecological and behavioral aspects in the region.

Acknowledgements

We would like to thank the Zegie district administers for allowing us to carry out this study in the Zegie Peninsula. Our thanks also go to the local people who answered the questionnaire survey politely..

References

1. Toledo VM. The biodiversity of Latin America: A threatened heritage. Ambiente y Desarrollo 1988; 4: 13–24.
2. Altieri MA, Toledo VM. The agroecological revolution in Latin America: Rescuing nature, ensuring food sovereignty and empowering peasants. J Peasant Stud. 2011; 38: 587–612.
3. Laurance W, Sayer J, Cassman K. Agricultural expansion and its impacts on tropical nature. Trends in Ecol Evol. 2014; 29: 107–116.
4. Fahrig L. Effects of habitat fragmentation on biodiversity. Annual Review of Ecol Evol Syst. 2003; 34: 487–515.
5. Pardini R, Souza SM, Braga-Neto R, Metzger JP. The role of forest structure, fragment size and corridors in maintaining small mammal abundance and diversity in an Atlantic forest landscape. Biol Conserv. 2005; 124: 253–266.
6. Harris JD, Brown PL. Wildlife; destruction, conservation and biodiversity. New York: Nova Science Publishers; 2009.
7. Husen A, Mishra V, Semwal K, Kumar D. Biodiversity status in Ethiopia and challenges. Environ Pollut Biodiver. 2012; 1: 31–79.
8. Awas T. Plant diversity in Western Ethiopia: Ecology, ethnobotany and conservation. Ph.D. Thesis; University of Oslo. 2007.
9. Yalden DW, Largen MJ. Endemic mammals of Ethiopia. Mammal Review. 2008; 22: 115–150.
10. Kingdon J. The Kingdon field guide to African mammals. Now York: Bloomsbury Publishing; 2015
11. Hoeck HN, Bloomer P. Rock Hyrax *Procavia capensis*. London: Bloomsbury Publishing; 2013.
12. Fairall N, Eloff AK, Mcnairn IS. Integration of metabolism and digestion in the hyrax. S Afr J Anim Sci. 1983; 13: 79–80.

13. Teklehaimanot G. Population ecology and threats of the rock hyrax (*Procavia Capensis*) in Bale Mountains National Park, Ethiopia. Zoological Sciences. Ph.D. Thesis, Addis Ababa University. 2015.
14. Stuart C, Stuart T. Field guide to the larger mammals of Africa. Cape Town: Struik Publishers; 2000.
15. Smith EA., Hill K, Marlowe FW, et al. Wealth transmission and inequality among hunter-gatherers. J curr Anthropol. 2010; 51: 1–16.
16. Nowak RM. Walker's mammals of the World, Baltimore, Maryland: The John Hopkins University Press; 1999.
17. Jacobson R, Eisenberger C, Svobodova M, et al. Outbreak of cutaneous leishmaniasis in Northern Israel. J Infect Dis. 2003; 188: 1065–1073.
18. Butynski T, Hoeck H, Koren L, De Jong YA. *Heterohyrax brucei*: The IUCN Red List of Threatened Species. 2015; e.T41766A2128587
19. Hoeck H, Klein H, Hoeck P, Flexible social organization in Hyrax1). Zeitschrift für Tierpsychologie. 1982; 59: 265–298.
20. Newmark W, Manyanza D, Gamassa D.-G, Sariko H. The conflict between wildlife and local people living adjacent to protected areas in Tanzania: Human density as a predictor. Conserv Biol. 1994: 8: 249–255.
21. Girma, T, Teshome Z, Mahari M, Knowledge, attitude and practices of peasants towards hyraxes in two selected church forests in Tigray Region, Northern Ethiopia. Int J Biodiv Conserv. 2015; 7: 299–307.
22. Asrat A. The tree hyrax (*Dendrohyrax arboreus*): Feeding behaviour, activity patterns and traditional medicinal use in Kafa Zone, Southwest Ethiopia. M.Sc. Thesis, Addis Ababa University. 2016.
23. Teklehaimanot G, Balakrishnan M, Distribution and population status of the endemic rock hyrax subspecies (*Procavia capensis capillosa*) of the Bale Mountains, Ethiopia. Int J Ecol Environ Sci. 2018; 44: 1–9.
24. Belay B, Zewdie S, Mekuria W, Abiyu A, Amare D, Woldemariam T. Woody species diversity and coffee production in remnant semi-natural dry Afromontane Forest in Zegie Peninsula, Ethiopia. Agrofor Syst. 2019; 93: 1793–1806.

25. Gebeyehu G, Bekele A. Human-wildlife conflict in Zegie Peninsula (Ethiopia) with emphasis on grivet monkey (*Cercopithecus aethiops aethiops*). SINET: Ethio J Sci. 2011; 32: 99–108.
26. Whittaker DG, Van Dyke WA, Love SL. Evaluation of aerial line transect for estimating ponghorn antelope abundance in low-density populations. Wildl Soc Bull. 2003; 31: 443–453.
27. Bowland A, Perrin M. Temporal and spatial patterns in blue duikers *Philatomba monticola* and red duikers *Cephalophus natalensis*. J Zool. 2009; 237, 487–498.
28. Barry R, Mundy P. Population dynamics of two species of hyraxes in the Matobo National Park, Zimbabwe. Afr J Ecol. 2002; 36: 221–233.
29. Young TP, Evans M.R. Alpine vertebrates of Mount Kenya, with particular notes on the rock hyrax. J East Afr Nat Hist. 1993; 82: 55–79.
30. Geist HJ. Lambin E.E. Proximate causes and underlying driving forces of tropical deforestation. BioScience, 2002; 52: 143–150.
31. Druce D, Brown J, Castley G, Kerley G, Kotler B, Slotow R, Knight M. Scale-dependent foraging costs: Habitat use by rock hyraxes (*Procavia capensis*) determined using giving-up densities. Oikos. 2006: 115: 513–525.
32. Parsons SDC, Smith SGD, Martins Q, et al. Pulmonary infection due to the dassie bacillus (*Mycobacterium tuberculosis* complex sp.) in a free-living dassie (rock hyrax - *Procavia capensis*) from South Africa. Tuberculosis, 2008; 88: 80–83.
33. Skinner JD, Chimimba CT. The mammals of the Southern African Sub-region. Cambridge: Cambridge University Press; 2005.
34. Franco PM. Factors affecting hyrax presence/absence and population size: A case study in Serengeti National Park, Tanzania. Norway: Norwegian University of Science and Technology; 2015.
35. Kershenbaum A, Kershenbaum A, Blaustein L. Rock hyrax (*Procavia capensis*) den site selection: Preference for artificial sites. Wildl Res. 2011; 38: 244–248.
36. Wiid RE, Butler HJB. Population management of rock hyraxes (*Procavia capensis*) in residential areas. Pest Manag Sci. 2015; 71: 180–188.
37. Kagoro-Rugunda G. Crop raiding around Lake Mburo National Park, Uganda. Afr J Ecol., 2004; 42: 32–41.

38. Kifle Z, Bekele A. Human–hamadryas baboon (*Papio hamadryas*) conflict in the Wonchit Valley, South Wollo, Ethiopia. Afr J Ecol. 2021; 59: 29–36.
39. Kifle Z. Human-olive baboon (*Papio anubis*) conflict in the human-modified landscape, Wollo, Ethiopia. Glob Ecol Conserv. 2021; 31: e01820.
40. Kifle Z, Bekele A. Human–gelada conflict and attitude of the local community toward the conservation of the southern gelada (*Theropithecus gelada obscurus*) around Borena Sayint National Park, Wollo, Ethiopia. Environ Manag. 2020; 65: 399–409.
41. Kifle Z, Assefa WW, Moges A. Human-hippo conflicts around Lake Tana Biosphere Reserve, Ethiopia: Vulnerability of hippopotamus in human-dominated landscape. PLoS ONE, 2023; 18: e0291802.
42. Power RJ. The Distribution and status of mammals in the North West Province. Department of economic development, environment, conservation and tourism, North West Provincial Government. South Africa: Mahikeng; 2014.

List of tables

Table 1. Population size and structure of the rock hyrax during the dry and wet seasons.

Habitat type	Season							
	Dry				Wet			
	Adult	Juvenile	Total	%	Adult	Juvenile	Total	%
Human residence	137	51	188	44	162	70	232	45
Forest	26	2	28	7	31	12	43	8
Lakeshore	157	53	210	49	166	71	237	46
Bushland	-	-	-		-	-	-	
Total	320	106	426	-	359	153	512	-

Table 2. Socioeconomic and demographic profile of the respondents.

Characteristics	Frequency	Percentage
Sex		
Male	105	51.7
Female	98	48.3
Age		
21-35	29	14.3
Above 35	174	85.7
Educational level		
Illiterate	59	29.1
Primary school	43	21.2
High school	31	15.3
Higher education level (above grade 12)	18	8.8
Religion (priests and deacons)	52	25,6

Table 3: Attitude of respondents towards rock hyrax.

Variables	Respondents' feeling	
	Good	Bad
Educational level		
Primary school	21 (48.8%)	22 (51.2%)
Secondary school	27 (90.0%)	3 (10.0%)
Higher education level (above grade 12)	19 (100%)	-
Religion (priests and deacons)	16 (30.8%)	36 (69.2%)
Uneducated	3 (5.1%)	56 (94.9%)
Total	*86 (42.4%)*	*117 (57.6)*
Gender		
Male	51 (48.6%)	54 (51.4%)
Female	14 (14.3%)	84 (85.7%)
Total	65 (32.0%)	138 (68.0%)

List of figure

Fig. 1. Conservation attitude towards rock hyrax in the region (religion = priests and deacons through informal education); (Higher education level = above grade 12).

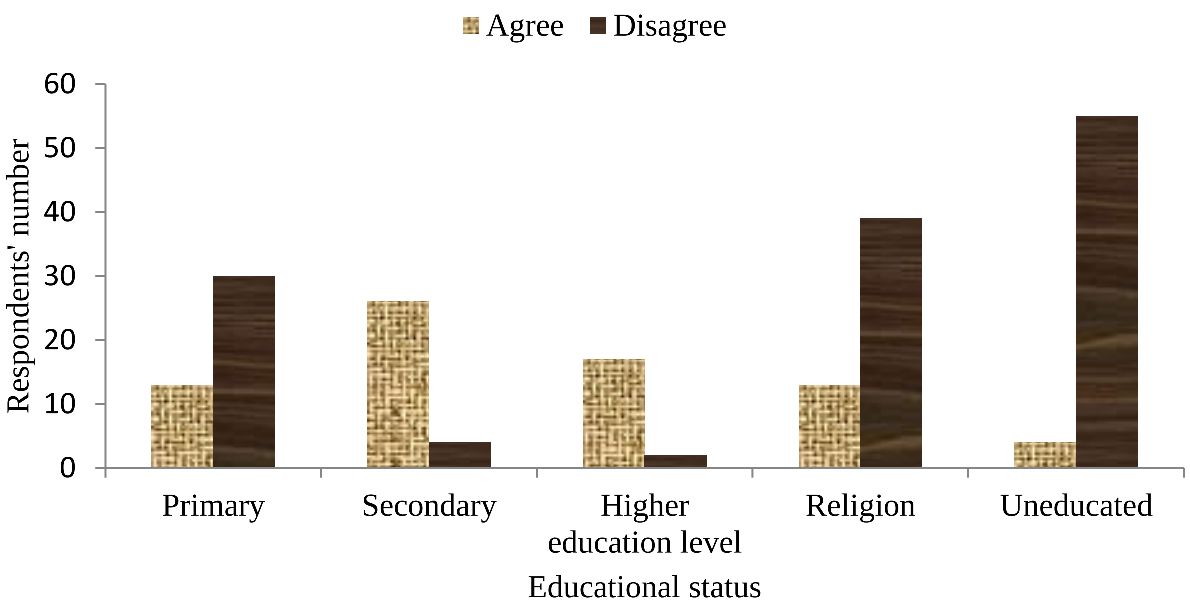

Population size, habitat association and local residents' attitude towards rock hyrax (*Procavia capensis*) in Zegie Peninsula, Ethiopia

Birkie Alehegn[1], Zewdu Kifle*[1]

[1]Department of Biology, Bahir Dar University, Bahir Dar, Ethiopia

Corresponding author: Zewdu Kifle, email: zewdu96@yahoo.com;

ORCID: https://orcid.org/0000-0002-0014-3525

Abstract

Given the current rate of habitat loss particularly in the tropics, reliable data on the population size and habitats of wild animals is crucial to initiate conservation and management activities in an area. Wildlife ecologists have not studied the ecology of most medium-sized mammals in detail ~~well~~. Rock hyrax (*Procavia capensis*) is one of the medium-sized mammals that ~~are~~ have not been well-studied in Ethiopia. Therefore, we conducted this study to determine the population size, and to examine the residents' attitude towards the rock hyrax in the Zegie Peninsula, Ethiopia. We applied the strip transect counting method to estimate the population size and determine the density of rock hyrax in the area. We used structured questionnaires and informant interviews to examine the attitude of local people towards rock hyrax ~~in the region~~. We counted ~~an average total population size of~~ 469 individuals of rock hyraxes in the study area. Out of this population, adults comprised 72.4% and juveniles comprised 27.6%. Through extrapolation, we estimated the total population of c. 2,184 rock hyraxes within the Zegie Peninsula. ~~We counted 426 individuals of rock hyrax during the dry season while we counted 512 individual during the wet season.~~ The ~~average~~ density of rock hyrax was 36.5 individuals/km^2. The ~~average~~ juvenile-to-adult age ratio was 1:2.6 in the ~~region~~ area. Most rock hyraxes were found in the lakeshore habitat followed by human residence area. Most respondents had negative attitudes towards the rock hyraxes. Respondents reported that rock hyraxes caused pollution in the church and resident compounds due to their pellets and urine. They also claimed that rock hyraxes ~~destructive~~ damaged ~~to the~~ fruits and vegetables ~~they harvest~~. The respondents traditionally believed that rock hyraxes have a magical power that takes food from the local people. Most respondents reported that rock hyraxes' body parts are used to prepare ~~for the preparation of~~ traditional medicines to cure human and cattle ailments. Key informants also

~~reported~~ stated that rock hyraxes' body parts are used to treat cattle anthrax, and their pellets are used to treat leprosy. Most respondents did not support the conservation of rock hyraxes in the area. Our results provide a baseline for ~~the~~ developing of conservation management strategies for the long-term survival of rock hyraxes ~~in the region~~.

Keywords: Conservation, population estimation, residents' attitude, rock hyrax, traditional medicine, Zegie Peninsula

Introduction

The biological diversity around the globe represents a resource that ~~offers~~ benefits ~~to~~ the current and future generations [1]. However, because of the need to feed a growing human population, forests have been cut down for agriculture, livestock grazing, settlement, and urbanization [2, 3]. This has led to the fragmentation of the landscape and has altered the distribution and abundance of ~~many~~ wild animals and plants [4]. The effect~~s~~ and magnitude of fragmentation on biodiversity ~~are~~ is complex [4, 5]. The human population in Africa is growing at a rapid rate which needs space to live and harvest. Human interference in the form of clearing animal habitats for agriculture has impacted African wildlife [6].

Ethiopia possesses different altitude ranges and topography [7]. The altitudinal variation of the country ranges from 126 meters below sea level in the Danakil Depression of the Afar region to the highest peak of 4,620 meters above sea level on Mount Ras Dejen of the Simien Mountains National Park. These variations in altitude and topography ~~variation~~ create diverse climatic conditions varying from the hot and dry desert in the lowland areas to cold and humid alpine habitats in the highlands. These factors ~~also~~ strongly influence Ethiopia's extraordinary range of terrestrial and aquatic ecosystems which have contributed to high flora and fauna diversity and rate of endemism [8, 9]. ~~Past geological history and wide-ranging climatic features have made Ethiopia the home of diverse endemic fauna and flora [9].~~

Hyraxes are medium-sized terrestrial mammals native to Africa and the Middle East. Hyrax comprises three extant genera: *Procavia*, *Heterohyrax*, and *Dendrohyrax*. Five extant species of hyraxes are recognized: the rock hyrax (*Procavia capensis*) and the yellow-spotted rock hyrax (*Heterohyrax brucei*), which both live on rock outcrops, including cliffs in Ethiopia and isolated granite outcrops called koppies in southern Africa; the western tree hyrax (*Dendrohyrax*

dorsalis), southern tree hyrax (*D. arboreus*), and eastern tree hyrax (*D. validus*). Their distribution is limited to Africa, except for *P. capensis* which is also found in the Middle East.

Rock hyraxes (*P. capensis*) look like a large guinea pig or a rabbit with rounded, short ears and no visible tail. The rock hyrax's coat color ranges ~~in color~~ from yellowish to grayish-brown, with a lighter underbelly. They have short legs very long bodies that are concealed by a hunched posture and long fur [10]. They have long, tactile hairs, especially on the muzzle, throat, cheeks, and rump. They eat mostly grass, fruits, and leaves [10, 11]. They have disproportionately large jaws and ~~an~~ the ability to draw the lips right back allowing them to bite off and chew large mouthfuls of vegetation at a time. Their habitats include the savanna, shrubland, desert, and rocky areas, such as cliffs and mountain peaks [10]. ~~Being,~~ Rock hyraxes are slow-moving ~~and~~ with only a few defensive mechanisms against predators. They depend on rock outcrops, which provide protection from predators, access to safe foraging areas, and good vantage points [12].

~~In Ethiopia,~~ Rock hyraxes (*P. capensis*) live in rock outcrops, piles of boulders, and fractured cliff faces [13]. ~~Rock hyrax~~ They have the widest geographical and altitudinal distribution among the hyraxes [14]. Even if there are no major threats to rock hyraxes, predation, intra-specific competition, immigration, territorial fighting, and dispersal regulate ~~rock hyrax~~ their population. ~~Rock hyraxes~~ They are hunted by people for their meat and soft fur. For example, the Hadza or Watindiga, a bushman tribe in Tanzania, hunt rock hyraxes for food [15]. In some areas of Egypt, they are ~~it is~~ hunted for food [16]. In countries like Kenya and Israel, ~~the~~ rock hyraxes are ~~is~~ not ~~supported~~ allowed to live in their localities for being ~~a~~ reservoirs of cutaneous *leishmaniasis* [17].

Rock hyrax is classified as a species of Least Concern Red List of Threatened Species [18] However; their populations have declined due to habitat loss, disease, and predation [19]. They are considered ~~as~~ a pest in some areas, competing with farmers by eating their crops. They are also subjected to threats from various avian and mammalian predators.

Assessing the population size of wild animals and their habitat association as well as understanding the attitudes of the local people towards the wild animal is important for designing sustainable conservation and management strategies [20]. Only a few studies have been conducted on the population size, distribution, and ecology of rock hyrax in Ethiopia [21, 22, 23]. Therefore ~~Thus~~, the population size and distribution of rock hyrax ~~is~~ are poorly known and documented in ~~Ethiopia~~ the country. Specifically, there is no information on the population size

of rock hyrax in the Zegie Peninsula, Ethiopia. In addition, the attitude of local people towards rock hyrax has not ~~been~~ studied ~~documented~~ in Ethiopia. Therefore, ~~this study is the first and~~ the objectives of the study were (1) to determine the population size of rock hyrax in the Zegie Peninsula**;** (2) to obtain information ~~determine~~ on the habitat association of rock hyrax in the area; and (3) to examine the attitude of local people towards rock hyrax conservation. Our findings provide baseline information ~~This study will be fundamental~~ for further studies (e.g., traditional medicine of rock hyrax and other ecological and behavioral aspects) in the area ~~region~~.

Materials and Methods

Ethical considerations

This study was carried out according to the guidelines of the review board of Bahir Dar University (BDU) of ~~the~~ human ethics research. The interviewer fully informed ~~to about~~ the respondents ~~on~~ the aim of the research and how the data obtained from them would be used. Confidentiality of information was assured by the investigator. Participants gave their consent verbally to participate and agreed after ~~being explained~~ the aim of the survey and how the interview would proceed. ~~Consent was obtained by having participants state that they to participate.~~

Study area

We carried out this study in the Zegie Peninsula, the southwestern shore of Lake Tana, Ethiopia. Zegie Peninsula covers 13.47 Km^2. The Zegie Peninsula is situated at 11°40' to 11°43' N latitude and 37°19' to 37°21' E. It is found at elevations between 1775 m and 1985 m above sea level. Zegie Peninsula is home to many Ethiopian Orthodox churches and monasteries dating back to the 13th century with a unique cultural, and religious heritage, and historical and aesthetic value. It is an important site for the persevering religion and culture of the country. It is one of the tourist destination sites in Ethiopia. The climate of Zegie Peninsula is a warm-temperate tropical highland monsoon with a mean temperature of 21.9°C. The area experiences two main seasons: the wet season and the dry season. The wet season typically occurs between June and October, while the dry season occurs between November and May.

The Peninsula is surrounded by Lake Tana in the northern and eastern directions, wetlands in its western parts, and farmlands in the southern directions. The dominant vegetation of the area ~~in this habitat~~ are *Mimusops kumme*, *Syzygium guineense*, *Ficus vasta*, ~~Shenbeko,~~ *Jasminum grandiflorum*, *Momordica foetida*, *Clematis simensis*, *Tragia brevipes*, *Urera hypselodendron and Phytolacca dodecandra*. The Zegie Peninsula is also home to different mammalian species like leopard (*Panthera pardus*), grivet monkey (*Chlorocebus aethiops*), rock hyrax (*Procavia capensis*), bushpig (*Potamochoerus larvatus*), and duiker (*Sylvicapra grimmia*). In addition, the peninsula supports diverse species of birds, amphibians, reptiles, mollusks, and insects. However, its wild animals and their habitats are under threat due to deforestation and illegal hunting practices [24]. For example, common bushbuck (*Tragelaphus scriptus*) and warthog (*Phacochoerus africanus*) have been eradicated due to illegal hunting in the area [25].

Population size and habitat association of rock hyraxes

Between January 10, 2021 and June 15, 2021, we applied a line transect method to estimate the population size of rock hyrax in the study area. The census zone was divided into several sample units. The selection of the sampling units was done by using two-stage sampling techniques. We divided the study area into four habitat strata: The lakeshore stratum of the coffee-dominated semi-natural forest, human premises, tall trees with coffee plantations, and bushland. We established ~~a total of~~ 18 transect lines in these four habitats. The average lengths of the line transects were 4.12 km in the human residence, 4.42 km in the forest, 1.83 km in the bushland, and 0.69 km in the forest. The distance between two consecutive transect lines was 200 to 300 m to avoid double counting ~~of rock hyraxes~~. Therefore, the possibility (likelihood) of double counting is almost zero using this distance gap between consecutive transects for the target species. The length and the number of transects were proportional to the size of ~~the~~ each habitat ~~type~~ stratum. During the transect survey, we recorded the number of rock hyrax seen, the age category, and the habitat type. We conducted transect walks for three consecutive days per month ~~from 3:30-5:30 hours~~ in the morning (9:00–11:30 a.m.) and ~~from 8:30-11:30 hours~~ in the late afternoon (8:30–11:00 p.m.) when the rock hyrax is active for foraging and sun basking at these day times. We surveyed transects by direct counting method walking at an average speed of 1km/hr [26]. Within the line transect, we moved along the lines, looking for the target species with a 25 m fixed transect width to the left and right sides of the line [27]. We paused two

minutes every 50 m to allow for the scanning of vegetation and rocky outcrops within the strip for the presence of the resting rock hyraxes. We sampled 2.71 km^2 from the total size of 12.62 km^2 area. The study covered the dry (February, March, and April) and wet (June, July, and September) seasons. We repeated the same transect during the dry and wet seasons and at different times of the day.

Questionnaire survey

Between March 16, 2021 and June 20, 2021, we used a structured questionnaire interview method to collect data on the attitude of local people towards rock hyrax. The questionnaire was pre-tested on a sample of 17 respondents to ensure its validity and reliability and to improve its clarity in the study area. We applied a two-stage sampling technique to select the respondents. First, we selected seven villages from two *kebeles* (i.e., Ura and Yiganda-Mehale Zegie) using purposive sampling. Second, we selected the household heads (respondents) from the sampling villages by systematic random sampling method using the recorded book ~~of each village~~ found in the *kebele* (local office). We interviewed ~~a total of~~ 210 respondents out of 1,116 households inhabiting the Peninsula, yielding a sampling intensity of 18.82%. However, we discarded seven questionnaires since they were not fully answered by the respondents. We designed the structured questionnaire to examine the attitudes, practices, and beliefs of the local people, knowledge of its significance on rock hyrax as well as to identify factors that influence community perceptions on rock hyraxes. The questionnaire also included respondents' level of education, age, sex, ~~educational status,~~ and livelihood. To triangulate the data collected by questionnaire, we undertook key informant interviews with nine dwellers (community leaders, elders, religious leaders, and association leaders) to express the culture and practice related to rock hyrax in the study area.

Data analysis

We summed up all transects of each habitat results to determine the total population of rock hyraxes [28]. We extrapolated the transect result to estimate the total population number of rock hyrax within the study area. We excluded bushland habitat from the analysis since we did not record any rock hyrax in this habitat during the study period. We determined the density of the rock hyrax population by dividing the counted population (individuals) in the sample sites by the

total area of the study area (km^2). We analyzed data using descriptive statistics, frequencies, and percentages. ~~We used ANOVA to examine the population size difference of rock hyraxes between the dry and wet seasons.~~ We used the Chi-square test of independence to test the hypothesis that categorical variables (gender vs. attitude towards rock hyrax as well as education level vs. attitude towards rock hyrax) are independent of each other. ~~to examine the attitude of local people towards rock hyrax based on respondents' educational background and sex.~~ We set the level of rejection for all tests ~~set~~ at α = 0.05.

Results

Population size of rock hyrax

We counted ~~a total mean of~~ 469 individuals of rock hyrax ~~individuals~~ in the study area ~~Zegie Peninsula~~. Out of this population, the number of adults was 339.5 (72.4%) and juveniles 129.5 (27.6%) ~~were juveniles~~. Through extrapolation, ~~we estimated~~ the total estimated population size of rock hyrax was 2184 individuals within the upper limit of 2648 and lower limit of 1720 with a 95% confidence interval of the difference ~~of rock hyrax~~ in the Zegie Peninsula. The density of rock hyrax was 37.2 individuals/km^2 in the Peninsula. The ~~average~~ adult-to-juvenile age ratio was 1:2.6.

From the total population, we counted 426 individuals of rock hyrax during the dry season and 512 during the wet season (Table 1). ~~The population size of rock hyrax did not show a significant difference between the wet and dry seasons ($F_{1,6}$ = 5.988, *P* = 0.068).~~ During the dry season, adults constituted 75.1%, ~~while~~ and babies (juveniles) 24.9 % ~~were babies (juveniles).~~ While during the wet season adults constituted 70.1% and babies 29.9% ~~were babies~~. Adult to juvenile ratio was 1:3.0 during the dry season ~~while the ratio was~~ and 1:2.3 during the wet season. The density of rock hyrax during the dry season was 33 individuals/km^2 and ~~it was~~ 40 individuals/km^2 during the wet season.

We recorded the highest numbers of rock hyrax in the lakeshore (223.5, 47.7%) and ~~while we recorded~~ the lowest ~~numbers of rock hyrax~~ in the forest habitat (35.5, 7.5%). We recorded 210 (44.8%) individuals in the human residence area (Table 1). We didn't record rock hyrax in the bushland habitat. We recorded the highest population size of rock hyraxes in the lakeshore habitat during the dry (210 individuals) and wet (237 individuals) seasons.

Questionnaire survey

Socio-demographic features of the respondents

We conducted the questionnaire interviews with 203 respondents of which 105 were males and 98 were females with ages ranging from 21 to 65 (Table 2). Most (97.5%) respondents reported that their livelihood depends on a coffee plantation, fruit production, fishing, wood (log) selling for firewood, small-scale trading, and handcraft making and selling. Most (97.0%) respondents had their farmlands. Most (71.4%) respondents did not have sheep while other ~~a few~~ (28.6%) respondents had sheep. A few (2.5%) respondents were governmental and non-governmental employees. Many (29.1%) respondents had not attended both formal and informal education.

Local residents' attitudes towards rock hyraxes

~~More than~~ Over half (57.6%) of respondents had negative attitudes towards rock hyraxes. On the contrary, other (42.4%) respondents had positive attitudes towards rock hyraxes ~~in their presence in the region~~ (Table 3). These positive feelings respondents expressed that they used rock hyrax as a food source for their domestic dogs and income by selling its hide and meat to traditional healers. Male respondents had ~~a~~ better positive feelings about the presence of rock hyraxes in the area than female respondents, and there ~~difference~~ was a statistically significant relationship between gender and attitudes towards rock hyraxes ($\chi2 = 27.37$, df = 1, $P = 0.001$). Higher levels of educational background respondent's had ~~a~~ better positive feelings about the presence of rock hyraxes. ~~than uneducated and religious education respondents and the difference was significant~~ The proportion of positive attitudes towards rock hyraxes among the respondents category of educational background was statistically significantly different ($\chi2 = 90.91$, df = 4, $P = 0.001$).

Most (64.5%) respondents disagreed with the idea of rock hyrax conservation in the area ~~region~~ while other (35.5%) respondents agreed with it ~~the idea of rock hyrax conservation~~. Most ~~of the~~ uneducated, religious, and primary school level respondents disagreed on rock hyraxes' protection, however; ~~but~~ many ~~of the~~ secondary school and ~~above~~ higher educational level respondents agreed on the conservation of rock hyraxes in their localities (Fig. 1). There was a statistically significant relationship between the level education and conservation attitude towards rock hyraxes ($\chi2 = 82.26$, df = 4, $P = 0.001$).

Perceived costs and benefits of rock hyrax presence

Most (80.0%) respondents reported that rock hyrax damaged fruits and vegetables. While a few (20%) respondents believed that rock hyrax did not cause significant crop damage in their localities. Similarly, the key informant interview participants deeply expressed the destructive behavior of rock hyrax on crops. In addition, many respondents reported that economically important plants like *Rhamnus prinoides*, *Carica papaya*, *Citrus lemon*, *Eucalyptus camaldulesis*, *Mimusops kummel*, and *Acacia pilispina* are damaged by rock hyraxes. Similarly, key informants also deeply expressed the destructive behavior of rock hyrax on plants.

Many local people had bad traditional myths about the rock hyraxes which they have ~~some kind of~~ a magic power (locally known as "*wosaji*"). They claimed that rock hyraxes take food magically ~~as~~ when they see people eating. In addition, the other common problem claimed by key informants was the occurrence of rock hyrax ~~feces~~ pellets and urine that created pollution (bad smell) in and around churches and resident ~~house~~ compounds.

Most (93.0%) respondents replied that rock hyraxes have ~~some~~ benefits. Of these respondents, 88.0% described that rock hyrax body parts are used ~~for the~~ to prepare ~~of~~ traditional medicines to cure ~~some~~ human and cattle ailments. While, a few (3.0%) respondents reported that the skin of rock hyrax is used as ~~traditional~~ medicine (specific diseases were not mentioned), and a few (2.0%) respondents used rock hyraxes for their dog's food. Key informants also reported the medicinal value of ~~that~~ rock hyrax in which its flesh is used to treat cattle anthrax and the ~~fecal~~ pellets are used to treat leprosy.

Awareness of rock hyrax population and conservation status

When we asked about the population size of rock hyraxes, 112 (55.0%) respondents reported that the population size has decreased in the area ~~region~~, 76 (37.0%) believed the number ~~it had~~ increased in recent years, and 15 (8.0%) had no ~~known~~ information ~~about the number of rock hyrax~~ whether they increased or decreased. Many (56.0%) respondents have sawed most frequently three individuals of rock hyraxes in a group while ~~and~~ other ~~respondents~~ (44.0%) respondents have sawed four to five individuals in ~~the~~ a group. Most ~~of the key~~ informants ~~interviewees~~ also informed that rock hyraxes were not seen as widely in lakeshores and ~~in~~ church compounds as they were seen years before. They reported that hunting for traditional medicine and dogs and habitat disturbances ~~and damage to shelters~~ like ~~big~~ cutting trees and stone fencing are responsible for their decrement in the area.

Controlling mechanisms

Many (57.0%) respondents used killing and other (43.0%) ~~of~~ respondents used chasing as a means of controlling crop damage caused by rock hyrax and avoiding pollution by its ~~fecal~~ pellets and urine. Local people killed rock hyrax with spears, poisons, traps, and dogs. They ~~also~~ chased away rock hyrax by throwing stones, ~~chasing~~ with dogs, and making noise.

Conservation threats to rock hyraxes

In the Zegie Peninsula, 115 (56.0%) respondents reported threats to rock hyraxes in their locality, while other (44.0%) respondents stated that ~~did not believe in the presence of~~ there is no threat to rock hyraxes ~~in the region~~. Of the 115 respondents who reported threats to rock hyraxes, most (65.2%) respondents considered hunting and killing ~~as~~ to be the main threat followed by predation by dogs (28.8%) and 6.0% human-based habitat disturbances.

Discussion

Most researchers do not study the population ~~ecology~~ and behavioral ecology of medium-sized mammals and their conflicts with local people. ~~Our~~ In the current study, we investigated the population size of rock hyrax ~~as well as~~ and the extent of human-rock hyrax conflict in the Zegie Peninsula, Ethiopia. We found ~~an average of~~ 469 individuals of rock hyraxes ~~populations~~ in the Peninsula. ~~From this total population of rock hyrax,~~ Adults constituted more ~~proportion~~ than ~~the~~ babies (juveniles). ~~Similarly, [13] recorded~~ The number of more adults ~~ratio~~ than babies is also recorded in the Bale Mountains National Park, Ethiopia [13]. This might be due to predation pressure on ~~the survival rate of adults more than~~ young individuals than adults. In addition, on many occasions babies might hide in their shelter causing them difficulty to be seen by the observers during transect walks.

In the present study, the average density of rock hyrax was 37.2 individuals/km^2. On the contrary, ~~[13] reported that~~ the mean population density of 664.5 and 529.9 individuals/km^2 during the wet and dry seasons was reported in the Bale Mountain National Park [13]. Rock hyrax population density studies elsewhere have also revealed a density of 20 to 100 individuals/ km^2 in Mount Kenya [29], 500 to 4,000 individuals/km^2 in the Serengeti National Park, Tanzania [19], and 73 to ~~and~~ 94 individuals/km^2 in the Matobo National Park, Zimbabwe [28]. The minimum availability of rock outcrops, cliffs with crevices, caves, and gorges having rock piles or crevices in the Zegie Peninsula might be the case for low population density ~~in the region~~. In

addition, high habitat disturbance in the current unprotected study area might be the cause for the minimum density of rock hyrax. Severe habitat alterations due to various human activities in the natural habitat influence the ecology and population dynamics of wild animals [30]. Natural habitat disturbance through human activities influences the population size and density of rock hyrax, the highest population size was recorded in areas where ~~there are~~ rock~~y~~ outcrops, cliffs, ~~and~~ grasses, and shrubs are available ~~in the vicinity of their shelter~~ [13]. It is also expected that protected areas like the Bale Mountains National Park, Ethiopia, and the Serengeti National Park, Tanzania accommodate a higher density of wild animals than a human-modified landscape like the current study area.

~~[11] found the~~ An extreme population fluctuation of hyrax is recorded at Montopos in Zimbabwe [11] and in the Serengeti National Park of Tanzania. [19]. ~~in National Park,~~ In Augrabies Falls National Park, South Africa, ~~it is also found that~~ hyrax populations fluctuate mainly due to predation, food availability, drought, and infectious disease [31]. In the present study, we counted more rock hyrax populations during the wet season than in the dry season. This might be the birth of more babies ~~pups~~ during the wet season when the availability of food that can satisfy their nutritional demands is higher ~~during this season~~. The wet season is full of ~~more~~ nutritious food items, which could promote the breeding of animals ~~and birth.~~ Hyrax population fluctuation between seasons is mainly due to predation~~, droughts,~~ and food availability ~~infectious diseases~~ [31, 32]. During the dry season, hyraxes expose themselves to greater predation risk as they seek food farther from thier covers and refuges [13, 31].

Rock hyraxes were not uniformly distributed in the study area. For example, we did not record rock hyrax in the bushland since this habitat might not be suitable for foraging and hiding ~~sites like rock outcrops and boulder piles.~~ However, we counted more rock hyraxes ~~around~~ in the lakeshore habitat. This might be due to the availability of ~~save and~~ abundant rock~~y~~ outcrops ~~for rock hyraxes~~ in this habitat to forage and shelter. Similarly, ~~[13] also documented that~~ rock hyrax is not uniformly distributed in the Bale Mountains National Park, Ethiopia [13]. The distribution of rock hyrax mainly depends on the availability of food and shelter that allows it to hide and watch its predators easily. ~~[29] also documented that~~ The distribution of rock hyraxes is delineated by the presence of appropriate shelter [29]. ~~Similarly, [33] reported that~~ Likewise rock hyraxes are typically associated with rock~~y~~ outcrops, cliffs, or piles of boulders with bushes [33]. ~~[34] stated that~~ The distribution of rock hyrax also decreases around human premises due to

habitat loss and human disturbance [34]. However, a significant number of rock hyraxes ~~also~~ dwelled around human dwellings in the current study area. This might be the availability the hiding places since local people make compound fences using rocks. These rock fences might help rock hyraxes from being attacked by potential predators. In addition, as animals have started to dwell in human-modified landscapes, they adapt to human disturbances in the area by using the available resources. This ecological and behavioral adaptation of rock hyraxes might help to roam and forage around human dwellings. However, further assessment of habitat characteristics is needed to confirm our justification in the present study area.

Most peop[illegible]id not like the presence of rock hyraxes in their locality. This might be due to the destructive behavior of fruits and vegetables. ~~causing pollution around churches and houses as they defecate or urinate, and~~ In addition, the traditional belief of the local people ~~that it had~~ on the rock hyrax's magic power to take their food might develop negative perceptions toward the animal. ~~they eat as it saw people when eating~~. Rock hyraxes are reservoirs of cutaneous *leishmaniasis* that can lead to disease outbreaks around human settlements [17, 35]. Members of rock hyrax colonies urinate and defecate in designated areas ~~(latrines)~~ that might cause bad smells in ~~an area~~ church and residence compounds. These behaviors of rock hyrax might ~~not be confortable for local people and~~ be the reasons that local people develop a negative attitude toward this animal. On the other hand, even though traditional medicine helps to promote conservation efforts, the local communities are bothered by the effects on the animals.

In the present study, respondents reported that rock hyrax damages crops. When rock hyraxes inhabit rock boulders or cliffs near farmlands they ~~heavily utilize~~ damage crop seedlings [13. 21]. ~~[36].~~ It is also documented that rock hyraxes inhabiting close to farmlands damage crops [36]. Similar studies in different parts of Africa also revealed that wild animals posed main ~~major~~ threats to crops and livestock [37, 38, 39]. ~~Baboons in particular are important crop raiders across much of their ranges in Ethiopia [38, 39].~~

A strong attitudinal difference was seen towards rock hyrax presence related to the educational level of respondents ~~background~~. Respondents of higher educational backgrounds had better feelings about rock hyrax ~~the~~ presence in their locality than uneducated and religious informal educational backgrounds. Moreover, males had a better positive feeling in ~~of~~ the presence of rock hyraxes in their locality than females. Crop damage and livestock predation by wild animals influence the attitude of local farmers toward their conservation [38, 39, 40, 41].

Most ~~illiterate~~ uneducated and primary school-level ~~educated~~ respondents disagreed with the idea of rock hyrax conservation. This indicated that most of the dwellers did not have ~~enough~~ awareness and had less concern for rock hyrax protection. ~~[42]~~ It is also noted that there have been concerns about the species ~~have been raised~~ by the landowners in the Magaliesberg province, North West Region of South Africa [42]. On the contrary, most of the secondary school and above educational level ~~educated and above~~ respondents agreed on the protection of rock hyraxes in the area ~~region~~. ~~This showed that~~ Therefore, formal education is ~~important~~ crucial to developing wildlife conservation initiatives in an area.

Conclusions

This study provides crucial information on the population size and habitat association of rock hyraxes ~~and local peoples' attitude towards~~ as well as the local peoples' attitude towards rock hyraxes and their ethnozoological importance ~~of this wild animal~~ ~~inhabiting the Zegie Peninsula, Ethiopia.~~ We identified the Peninsula as an important site for the conservation of rock hyrax. However, human disturbances, mainly ~~with~~ the absence of enough shelter, ~~in the area~~ affect the population density of rock hyrax in the area.

Even if rock hyraxes damaged crops, local people use rock hyraxes as a traditional medicine to treat human and livestock aliments. This practice is important to promote conservation measures for rock hyrax in Ethiopia. The local administration and other governmental and non-governmental organizations should work cooperatively to create awareness and educate the local community about the economic and ecological importance and conservation of rock hyrax. We recommend that local people grow cash crops like coffee, mango, and avocado in the area to reduce conflicts between the local people and rock hyraxes regarding crop damage. Local people should create alternative job opportunities like fish pond farming and livestock ~~fattening~~ to benefit economically. In addition it is crucial to promote the medicinal values of rock hyraxes among the local community and other stakeholders. These actions help as ~~the~~ habitat management strategies to minimize the rock hyrax habitat from further alternations and destructions. These strategies also help to balance the needs of both the animals and the people. ~~This~~ study provides baseline information for further studies on ~~traditional~~ the medicinal values of rock hyrax as well as ecological and behavioral aspects in the region.

Acknowledgements

~~This work was funded by the Bahir Dar University.~~ We would like to thank the Zegie district administers for allowing us to carry out this study in the Zegie Peninsula. Our thanks also go to the local people who answered the questionnaire survey politely.

References

1. Toledo VM. The biodiversity of Latin America: A threatened heritage. Ambiente y Desarrollo 1988; 4: 13–24.
2. Altieri MA, Toledo VM. The agroecological revolution in Latin America: Rescuing nature, ensuring food sovereignty and empowering peasants. J Peasant Stud. 2011; 38: 587–612.
3. Laurance W, Sayer J, Cassman K. Agricultural expansion and its impacts on tropical nature. Trends in Ecol Evol. 2014; 29: 107–116.
4. Fahrig L. Effects of habitat fragmentation on biodiversity. Annual Review of Ecol Evol Syst. 2003; 34: 487–515.
5. Pardini R, Souza SM, Braga-Neto R, Metzger JP. The role of forest structure, fragment size and corridors in maintaining small mammal abundance and diversity in an Atlantic forest landscape. Biol Conserv. 2005; 124: 253–266.
6. Harris JD, Brown PL. Wildlife; destruction, conservation and biodiversity. New York: Nova Science Publishers; 2009.
7. Husen A, Mishra V, Semwal K, Kumar D. Biodiversity status in Ethiopia and challenges. Environ Pollut Biodiver. 2012; 1: 31–79.
8. Awas T. Plant diversity in Western Ethiopia: Ecology, ethnobotany and conservation. Ph.D. Thesis; University of Oslo. 2007.
9. Yalden DW, Largen MJ. Endemic mammals of Ethiopia. Mammal Review. 2008; 22: 115–150.
10. Kingdon J. The Kingdon field guide to African mammals. Now York: Bloomsbury Publishing; 2015
11. Hoeck HN, Bloomer P. Rock Hyrax *Procavia capensis*. London: Bloomsbury Publishing; 2013.
12. Fairall N, Eloff AK, Mcnairn IS. Integration of metabolism and digestion in the hyrax. S Afr J Anim Sci. 1983; 13: 79–80.

13. Teklehaimanot G. Population ecology and threats of the rock hyrax (*Procavia Capensis*) in Bale Mountains National Park, Ethiopia. Zoological Sciences. Ph.D. Thesis, Addis Ababa University. 2015.
14. Stuart C, Stuart T. Field guide to the larger mammals of Africa. Cape Town: Struik Publishers; 2000.
15. Smith EA., Hill K, Marlowe FW, et al. Wealth transmission and inequality among hunter-gatherers. J curr Anthropol. 2010; 51: 1–16.
16. Nowak RM. Walker's mammals of the World, Baltimore, Maryland: The John Hopkins University Press; 1999.
17. Jacobson R, Eisenberger C, Svobodova M, et al. Outbreak of cutaneous leishmaniasis in Northern Israel. J Infect Dis. 2003; 188: 1065–1073.
18. Butynski T, Hoeck H, Koren L, De Jong YA. *Heterohyrax brucei*: The IUCN Red List of Threatened Species. 2015; e.T41766A2128587
19. Hoeck H, Klein H, Hoeck P, Flexible social organization in Hyrax1). Zeitschrift für Tierpsychologie. 1982; 59: 265–298.
20. Newmark W, Manyanza D, Gamassa D.-G, Sariko H. The conflict between wildlife and local people living adjacent to protected areas in Tanzania: Human density as a predictor. Conserv Biol. 1994: 8: 249–255.
21. Girma, T, Teshome Z, Mahari M, Knowledge, attitude and practices of peasants towards hyraxes in two selected church forests in Tigray Region, Northern Ethiopia. Int J Biodiv Conserv. 2015; 7: 299–307.
22. Asrat A. The tree hyrax (*Dendrohyrax arboreus*): Feeding behaviour, activity patterns and traditional medicinal use in Kafa Zone, Southwest Ethiopia. M.Sc. Thesis, Addis Ababa University. 2016.
23. Teklehaimanot G, Balakrishnan M, Distribution and population status of the endemic rock hyrax subspecies (*Procavia capensis capillosa*) of the Bale Mountains, Ethiopia. Int J Ecol Environ Sci. 2018; 44: 1–9.
24. Belay B, Zewdie S, Mekuria W, Abiyu A, Amare D, Woldemariam T. Woody species diversity and coffee production in remnant semi-natural dry Afromontane Forest in Zegie Peninsula, Ethiopia. Agrofor Syst. 2019; 93: 1793–1806.

25. Gebeyehu G, Bekele A. Human-wildlife conflict in Zegie Peninsula (Ethiopia) with emphasis on grivet monkey (*Cercopithecus aethiops aethiops*). SINET: Ethio J Sci. 2011; 32: 99–108.
26. Whittaker DG, Van Dyke WA, Love SL. Evaluation of aerial line transect for estimating ponghorn antelope abundance in low-density populations. Wildl Soc Bull. 2003; 31: 443–453.
27. Bowland A, Perrin M. Temporal and spatial patterns in blue duikers *Philatomba monticola* and red duikers *Cephalophus natalensis*. J Zool. 2009; 237, 487–498.
28. Barry R, Mundy P. Population dynamics of two species of hyraxes in the Matobo National Park, Zimbabwe. Afr J Ecol. 2002; 36: 221–233.
29. Young TP, Evans M.R. Alpine vertebrates of Mount Kenya, with particular notes on the rock hyrax. J East Afr Nat Hist. 1993; 82: 55–79.
30. Geist HJ. Lambin E.E. Proximate causes and underlying driving forces of tropical deforestation. BioScience, 2002; 52: 143–150.
31. Druce D, Brown J, Castley G, Kerley G, Kotler B, Slotow R, Knight M. Scale-dependent foraging costs: Habitat use by rock hyraxes (*Procavia capensis*) determined using giving-up densities. Oikos. 2006: 115: 513–525.
32. Parsons SDC, Smith SGD, Martins Q, et al. Pulmonary infection due to the dassie bacillus (*Mycobacterium tuberculosis* complex sp.) in a free-living dassie (rock hyrax - *Procavia capensis*) from South Africa. Tuberculosis, 2008; 88: 80–83.
33. Skinner JD, Chimimba CT. The mammals of the Southern African Sub-region. Cambridge: Cambridge University Press; 2005.
34. Franco PM. Factors affecting hyrax presence/absence and population size: A case study in Serengeti National Park, Tanzania. Norway: Norwegian University of Science and Technology; 2015.
35. Kershenbaum A, Kershenbaum A, Blaustein L. Rock hyrax (*Procavia capensis*) den site selection: Preference for artificial sites. Wildl Res. 2011; 38: 244–248.
36. Wiid RE, Butler HJB. Population management of rock hyraxes (*Procavia capensis*) in residential areas. Pest Manag Sci. 2015; 71: 180–188.
37. Kagoro-Rugunda G. Crop raiding around Lake Mburo National Park, Uganda. Afr J Ecol., 2004; 42: 32–41.

38. Kifle Z, Bekele A. Human–hamadryas baboon (*Papio hamadryas*) conflict in the Wonchit Valley, South Wollo, Ethiopia. Afr J Ecol. 2021; 59: 29–36.
39. Kifle Z. Human-olive baboon (*Papio anubis*) conflict in the human-modified landscape, Wollo, Ethiopia. Glob Ecol Conserv. 2021; 31: e01820.
40. Kifle Z, Bekele A. Human–gelada conflict and attitude of the local community toward the conservation of the southern gelada (*Theropithecus gelada obscurus*) around Borena Sayint National Park, Wollo, Ethiopia. Environ Manag. 2020; 65: 399–409.
41. Kifle Z, Assefa WW, Moges A. Human-hippo conflicts around Lake Tana Biosphere Reserve, Ethiopia: Vulnerability of hippopotamus in human-dominated landscape. PLoS ONE, 2023; 18: e0291802.
42. Power RJ. The Distribution and status of mammals in the North West Province. Department of economic development, environment, conservation and tourism, North West Provincial Government. South Africa: Mahikeng; 2014.

List of tables

Table 1. Population size and structure of the rock hyrax during the dry and wet seasons.

Habitat type	Season							
	Dry				Wet			
	Adult	Juvenile	Total	%	Adult	Juvenile	Total	%
Human residence	137	51	188	44	162	70	232	45
Forest	26	2	28	7	31	12	43	8
Lakeshore	157	53	210	49	166	71	237	46
Bushland	-	-	-		-	-	-	
Total	320	106	426	-	359	153	512	-

Table 2. Socioeconomic and demographic profile of the respondents.

Characteristics	Frequency	Percentage
Sex		
Male	105	51.7
Female	98	48.3
Age		
21-35	29	14.3
Above 35	174	85.7
Educational level		
Illiterate	59	29.1
Primary school	43	21.2
High school	31	15.3
Higher education level (above grade 12)	18	8.8
Religion (priests and deacons)	52	25,6

Table 3: Attitude of respondents towards rock hyrax.

Variables	Respondents' feeling	
	Good	Bad
Educational level		
Primary school	21 (48.8%)	22 (51.2%)
Secondary school	27 (90.0%)	3 (10.0%)
Higher education level (above grade 12)	19 (100%)	-
Religion (priests and deacons)	16 (30.8%)	36 (69.2%)
Uneducated	3 (5.1%)	56 (94.9%)

Total	*86 (42.4%)*	*117 (57.6)*
Gender		
Male	51 (48.6%)	54 (51.4%)
Female	14 (14.3%)	84 (85.7%)
Total	65 (32.0%)	138 (68.0%)

~~Educational level~~	~~Respondents' feeling~~		~~Total~~
	~~Good~~	~~Bad~~	
~~Primary (1-8)~~	~~21 (10.0%)~~	~~22 (11%)~~	~~43 (21.0%)~~
~~Secondary (9-12)~~	~~27 (14.0%)~~	~~3 (1.0)~~	~~30 (15.0%)~~
~~Certificate and above~~	~~19 (9.0%)~~	~~-~~	~~19 (9.0%)~~
~~Religion~~	~~16 (8.0%)~~	~~36 (18.0%)~~	~~52 (26.0%)~~
~~Illiterate~~	~~3 (1.0%)~~	~~56 (28.0%)~~	~~59 (29.0 %)~~

List of figure

Fig 1. Conservation attitude ~~of~~ towards rock hyrax in the region (religion = priests and deacons through informal education); (certificate and above = above grade 12).

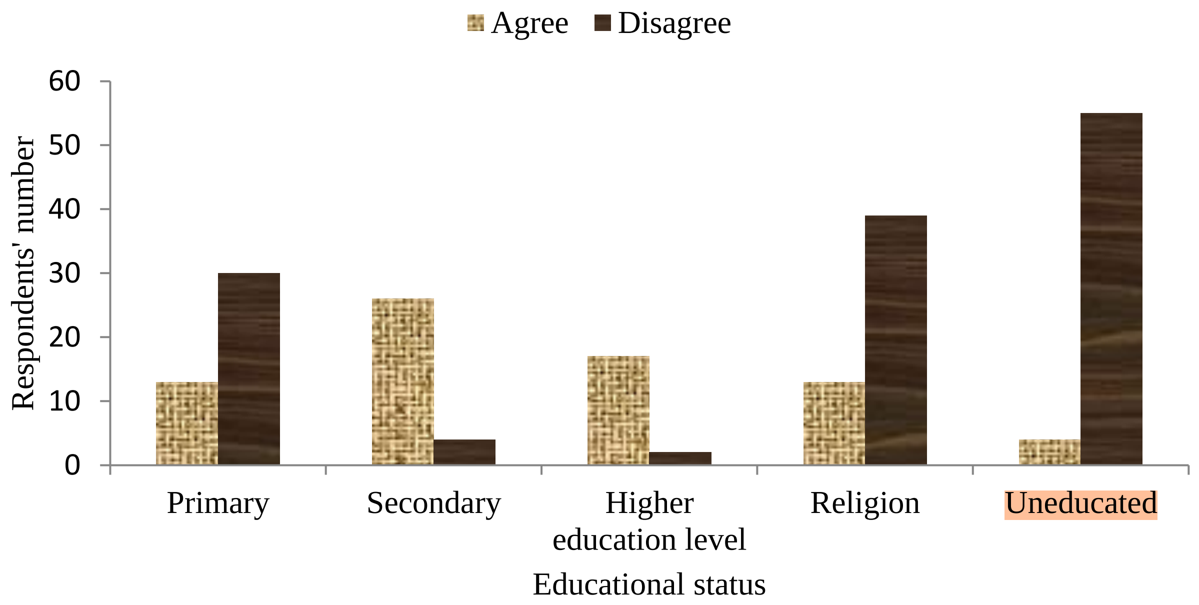

09 November 2024

Ethiopia

Ref.:
PONE-D-24-32415
Dear Editor in Chief

PLOS ONE

This is a revision of our manuscript entitled "**Population size, habitat association and local residents' attitude towards rock hyrax (*Procavia capensis*) in Zegie Peninsula, Ethiopia**" by Birkie Alehegn and Zewdu Kifle. On 15 October 2024, we received a major revision request to be acceptable for publication. You invited us to resubmit the revised version of the manuscript that addressed the points raised during the review process.

Hence, we addressed all of the reviewers' comments within this resubmitted manuscript.

Below there are lists of the revisions.

Sincerely,

Zewdu Kifle,

corresponding author

Response for Editor and reviewers

Thank you for your critical comments. I highlighted revisions with green color. In addition, I highlighted with red color and strikethrough those deleted letters, words, phrases, or sentences. In addition, I provided reviewers' concerns with justification within this file.

Thank you!

Response for Editor

Thank you! I have revised in detail on the presentation of this manuscript.

Response for reviewers

Reviewer #1:

Thank you for your important comments.

1. Population estimation methodology

Thank you! We determined the lengths and the numbers of transects in proportion to the size of the habitat strata. We repeated the same transects at each time of the day and season. We clarified why we have chosen those specific times of the day as the rock hyrax is mostly active for foraging and sun basking at these times of the day. We stated this in the revised version of the manuscript. We included confidence intervals.

2. Data on habitat association

   Thank you! We noted why we did not record rock hyraxes in the bushland habitat. This habitat might not be suitable for foraging and hiding sites like rock outcrops. However, a habitat preference index or another statistical tool for the animal requires more habitat characteristics data. This parameter needs additional research to answer the question in more detail. Thus, we recommended to do further ecological research in the area
3. Questionnaire design and analysis

   Thank you again! We performed a preliminary survey to ensure validity and reliability in the area. This pilot test was checked through data collection from 17 respondents. We stated the information in this revised version of the manuscript. We analyzed the data using the Chi-square test of independence to relate educational level and other factors to attitudes
4. Lack of ecological context and comparison

   Food availability, better hiding places, and high human disturbances are considered factors for the low density of rock hyrax in the present study area compared to protected areas like the Bale Mountain National Park in Ethiopia and Serengeti National Park in Tanzania. We have stated such ecological aspects in the discussion section.
5. Cultural considerations

   Thank you! It is very a fascinating cultural belief of the local community about the rock hyrax. That is why about 42.4% of the respondents had a good attitude towards rock hyrax in the study area. However, still more respondents had negative attitudes toward the animal. Yes, you are right. The traditional medicine value of the rock hyrax helps to promote conservation efforts in the region.
6. Conservation recommendations

   Thank you! We added actionable recommendations to balance the needs of both the animals and the local people in the area

Reviewer #2

General comments:

Thank you for your critical comments on this research work.

1. Thank you for appreciating the usefulness of the study.
2. Thank you! We corrected all reference citations.
3. Thank you we deleted this local name of rock hyrax ‘Shembeko’

4. Thank you here again! To avoid double counting, we counted consecutive transects simultaneously. The distance between two consecutive transect lines was 200 to 300 m to avoid double counting. Therefore the likelihood of double counting is almost zero. We scanned the habitat of rock hyrax within the strip with brief pauses of two minutes every 50 m. We stated this in the revised version of the manuscript.
5. Thank you! Sorry, we used the Ethiopian time zone. We corrected it.
6. We put the overall mean number of rock hyraxes of each habitat stratum.
7. Thank you! We added the reason why we counted a high proportion (44/45%) of rock hyraxes in the human dwellings in the discussion section.
8. In tables 2 &3 religion, these are deacons and priests who learned religion through the Ethiopian Orthodox Church. We designed respondents who completed grade 12 as certificates and above.

Reviewer #3:
Thank you for appreciating the manuscript's information. I corrected concerns that you raised in the annotated pdf.

Specific comments:

1. Line 11: Thank you! We changed the word 'well' with 'in detail'.
2. Line 27 and 33. We changed the word 'region' into area
3. Line 101 and 102: Thank toy! We rewrote the sentences.
4. Line 16: We deleted 'Shenbeko'.
5. Line 168: We deleted 'a total mean of' and we changed 'Zegie Peninsula' with study 'area'.
6. Line 191: Thank you! We added 'a' before 'few'.
7. Line 195-201: We edited based on your comment.
8. Line 204-205: We corrected
9. Line 219: We deleted 'some' from the sentence.
10. Line 229-220: We edited the sentence.
11. Line 247: Thank you! We corrected the sentence based on your comments.
12. Line 246: We edited the phrase 'habitat disturbance'.
13. Line 251-254: we corrected those sentences. We put the reference '13' as the end of the sentence.
14. Lien 256: We put the reference at the end of the sentence.
15. Line 259: We corrected it.
16. Line 262: We deleted 'in the region'.
17. Line 267: We edited it.
18. Line 269 and 270: We put these references at the end of the sentence.
19. Line 280: We deleted 'save'
20. Line 282, 284, 285, and 287: We put the reference at the end of the sentence.
21. Line 286: We deleted letter 'y' from rocky.

22. Line 292: We edited this sentence.
23. Line 295: Thank you! We edited this sentence.
24. Line 297 and 298: We edited these sentences.
25. Line 298: We put the reference at the end of the sentence.
26. Line 300-301: We deleted this sentence.
27. Line 303: We deleted the word ‘background’.
28. Line 309: We deleted the word ‘enough’.
29. Line 310: We put the reference at the end of the sentence.
30. Line 312: We corrected this sentence.
31. Line 317: Thank you! We corrected this sentence.
32. Line 332: We corrected this sentence.
