# Supplementary material for: Population size, habitat association, and local residents’ attitude towards rock hyrax (Procavia capensis) in Zegie Peninsula, Ethiopia
Source: PLoS One. 2025 Jun 30;20(6):e0323878. doi: 10.1371/journal.pone.0323878 (PMC12208434; doi:10.1371/journal.pone.0323878)
Supplement: Attachment — Submitted filename: Zewdu Kifle_Response to Reviewers_PLOS ONE.docx [file pone.0323878.s008.docx]

09 November 2024

Ethiopia

Ref.:

PONE-D-24-32415

Dear Editor in Chief

**PLOS ONE**

This is a revision of our manuscript entitled “**Population size, habitat association and local residents’ attitude towards rock hyrax (*Procavia capensis*) in Zegie Peninsula, Ethiopia**" by Birkie Alehegn and Zewdu Kifle. On 15 October 2024, we received a major revision request to be acceptable for publication. You invited us to resubmit the revised version of the manuscript that addressed the points raised during the review process.

Hence, we addressed all of the reviewers’ comments within this resubmitted manuscript.

Below there are lists of the revisions.

Sincerely,

Zewdu Kifle,

corresponding author

**Response for Editor and reviewers**

Thank you for your critical comments. I highlighted revisions with green color. In addition, I highlighted with red color and strikethrough those deleted letters, words, phrases, or sentences. In addition, I provided reviewers’ concerns with justification within this file.

Thank you!

**Response for Editor**

Thank you! I have revised in detail on the presentation of this manuscript.

**Response for reviewers**

**Reviewer #1:**

Thank you for your important comments.

1. Population estimation methodology

Thank you! We determined the lengths and the numbers of transects in proportion to the size of the habitat strata. We repeated the same transects at each time of the day and season. We clarified why we have chosen those specific times of the day as the rock hyrax is mostly active for foraging and sun basking at these times of the day. We stated this in the revised version of the manuscript. We included confidence intervals.

2. Data on habitat association

Thank you! We noted why we did not record rock hyraxes in the bushland habitat. This habitat might not be suitable for foraging and hiding sites like rock outcrops. However, a habitat preference index or another statistical tool for the animal requires more habitat characteristics data. This parameter needs additional research to answer the question in more detail. Thus, we recommended to do further ecological research in the area

3. Questionnaire design and analysis

Thank you again! We performed a preliminary survey to ensure validity and reliability in the area. This pilot test was checked through data collection from 17 respondents. We stated the information in this revised version of the manuscript. We analyzed the data using the Chi-square test of independence to relate educational level and other factors to attitudes

4. Lack of ecological context and comparison

Food availability, better hiding places, and high human disturbances are considered factors for the low density of rock hyrax in the present study area compared to protected areas like the Bale Mountain National Park in Ethiopia and Serengeti National Park in Tanzania. We have stated such ecological aspects in the discussion section.

5. Cultural considerations

Thank you! It is very a fascinating cultural belief of the local community about the rock hyrax. That is why about 42.4% of the respondents had a good attitude towards rock hyrax in the study area. However, still more respondents had negative attitudes toward the animal. Yes, you are right. The traditional medicine value of the rock hyrax helps to promote conservation efforts in the region.

6. Conservation recommendations

Thank you! We added actionable recommendations to balance the needs of both the animals and the local people in the area

**Reviewer #2**

**General comments:**

Thank you for your critical comments on this research work.

1. Thank you for appreciating the usefulness of the study.
2. Thank you! We corrected all reference citations.
3. Thank you we deleted this local name of rock hyrax ‘Shembeko’
4. Thank you here again! To avoid double counting, we counted consecutive transects simultaneously. The distance between two consecutive transect lines was 200 to 300 m to avoid double counting. Therefore the likelihood of double counting is almost zero. We scanned the habitat of rock hyrax within the strip with brief pauses of two minutes every 50 m. We stated this in the revised version of the manuscript.
5. Thank you! Sorry, we used the Ethiopian time zone. We corrected it.
6. We put the overall mean number of rock hyraxes of each habitat stratum.
7. Thank you! We added the reason why we counted a high proportion (44/45%) of rock hyraxes in the human dwellings in the discussion section.
8. In tables 2 &3 religion, these are deacons and priests who learned religion through the Ethiopian Orthodox Church. We designed respondents who completed grade 12 as certificates and above.

**Reviewer #3:**

Thank you for appreciating the manuscript’s information. I corrected concerns that you raised in the annotated pdf.

**Specific comments:**

1. Line 11: Thank you! We changed the word ‘well’ with ‘in detail’.
2. Line 27 and 33. We changed the word ‘region’ into area
3. Line 101 and 102: Thank toy! We rewrote the sentences.
4. Line 16: We deleted ‘Shenbeko’.
5. Line 168: We deleted ‘a total mean of’ and we changed ‘Zegie Peninsula’ with study ‘area’.
6. Line 191: Thank you! We added ‘a’ before ‘few’.
7. Line 195-201: We edited based on your comment.
8. Line 204-205: We corrected
9. Line 219: We deleted ‘some’ from the sentence.
10. Line 229-220: We edited the sentence.
11. Line 247: Thank you! We corrected the sentence based on your comments.
12. Line 246: We edited the phrase ‘habitat disturbance’.
13. Line 251-254: we corrected those sentences. We put the reference ‘13’ as the end of the sentence.
14. Lien 256: We put the reference at the end of the sentence.
15. Line 259: We corrected it.
16. Line 262: We deleted ‘in the region’.
17. Line 267: We edited it.
18. Line 269 and 270: We put these references at the end of the sentence.
19. Line 280: We deleted ‘save’
20. Line 282, 284, 285, and 287: We put the reference at the end of the sentence.
21. Line 286: We deleted letter ‘y’ from rocky.
22. Line 292: We edited this sentence.
23. Line 295: Thank you! We edited this sentence.
24. Line 297 and 298: We edited these sentences.
25. Line 298: We put the reference at the end of the sentence.
26. Line 300-301: We deleted this sentence.
27. Line 303: We deleted the word ‘background’.
28. Line 309: We deleted the word ‘enough’.
29. Line 310: We put the reference at the end of the sentence.
30. Line 312: We corrected this sentence.
31. Line 317: Thank you! We corrected this sentence.
32. Line 332: We corrected this sentence.
