# Supplementary material for: Population size, habitat association, and local residents’ attitude towards rock hyrax (Procavia capensis) in Zegie Peninsula, Ethiopia
Source: PLoS One. 2025 Jun 30;20(6):e0323878. doi: 10.1371/journal.pone.0323878 (PMC12208434; doi:10.1371/journal.pone.0323878)
Supplement: Attachment — Submitted filename: Recommendation for improvment.docx [file pone.0323878.s007.docx]

Still the manuscript needs to address the following weakness

**1. Originality of Research**

- **Strengths**: The manuscript presents original research on the diversity and habitat association of medium and large mammals in the Dhidhessa Wildlife Sanctuary (DWS), Southwestern Ethiopia. The study provides new data on species composition, abundance, and habitat preferences, which are not previously documented for this specific area.
- **Weaknesses**: While the study is original, it does not explicitly state how it differs from or builds upon previous studies in similar regions (e.g., Gambella National Park, Dhati Wolel National Park). A more detailed comparison with existing literature would strengthen the originality claim.

**2. Results Not Published Elsewhere**

- **Strengths**: The manuscript does not indicate that the results have been published elsewhere. The data collection period (December 2022 to June 2023) suggests that the findings are recent and unique to this study.
- **Weaknesses**: There is no explicit statement confirming that the results have not been published elsewhere. Including such a statement would clarify this criterion.

**3. Technical Standard and Detail of Methods**

- **Strengths**: The study employs a variety of methods, including line transect surveys, camera trapping, and indirect evidence (e.g., scats, footprints), which are appropriate for assessing mammal diversity and habitat association. The use of stratified random sampling and the inclusion of both wet and dry seasons add rigor to the study design.
- **Weaknesses**:
  - The manuscript lacks detailed descriptions of some methods. For example, the specific models and settings of the camera traps are not mentioned, which could affect the reproducibility of the study.
  - The statistical analysis is described briefly, with no detailed explanation of how the chi-square test and Shannon-Wiener Diversity Index were applied. More detail on the statistical methods and software settings would improve transparency.
  - The sample size (25 transects covering 24.65 km²) is relatively small for a sanctuary of 1300 km². The authors should justify why this sample size is sufficient and discuss potential limitations.

**4. Appropriate Conclusions Supported by Data**

- **Strengths**: The conclusions are generally supported by the data. The study identifies 27 mammalian species, with Artiodactyla and Carnivora being the most abundant orders. The findings on habitat preferences (e.g., riparian forests supporting the highest species diversity) are well-supported by the data.
- **Weaknesses**:
  - The discussion could be more critical. For example, the authors attribute the lower diversity of mammals in DWS compared to other parks to anthropogenic activities but do not provide direct evidence or data to support this claim.
  - The conclusion that "food, water, and protection" are decisive factors in species distribution is somewhat speculative. The study does not directly measure these variables or their impact on species distribution.

**5. Intelligibility and Language**

- **Strengths**: The manuscript is generally well-written and intelligible. The structure follows a logical flow, and the language is mostly clear.
- **Weaknesses**:
  - There are occasional grammatical errors and awkward phrasing (e.g., "The study revealed that high diversity of species in the area including the rare and threatened species"). These should be corrected for clarity.
  - Some sections, such as the methods, could benefit from more precise language. For example, the description of the transect lines and camera trapping could be more detailed.

**6. Ethics of Experimentation and Research Integrity**

- **Strengths**: The study involves non-invasive methods (e.g., camera trapping, indirect evidence), which align with ethical standards for wildlife research. The authors state that no approval from research ethics committees was required, which is reasonable given the nature of the study.
- **Weaknesses**:
  - The manuscript does not explicitly address potential ethical concerns related to human-wildlife conflict or the impact of the study on local communities. Given the mention of anthropogenic activities, a brief discussion of ethical considerations related to human interactions with wildlife would be beneficial.

**7. Adherence to Reporting Guidelines and Data Availability**

- **Strengths**: The manuscript includes a data availability statement confirming that all relevant data are within the paper and its supporting information files. This aligns with PLOS ONE's data policy.
- **Weaknesses**:
  - The manuscript does not mention adherence to specific reporting guidelines (e.g., STROBE for observational studies). Including a statement on adherence to such guidelines would improve transparency.
  - While the data availability statement is present, it is vague. Providing more specific details (e.g., where the supporting information files can be accessed) would enhance reproducibility.

**Overall Assessment:**

The manuscript presents valuable and original research on mammal diversity and habitat association in the Dhidhessa Wildlife Sanctuary. However, several areas need improvement to meet PLOS ONE's publication standards:

1. **Methodological Detail**: Provide more detailed descriptions of the methods, especially regarding camera trapping and statistical analysis.
2. **Critical Discussion**: Strengthen the discussion by addressing limitations and providing more direct evidence for conclusions.
3. **Language and Clarity**: Address grammatical errors and improve the clarity of certain sections, particularly the methods.
4. **Ethical Considerations**: Briefly discuss ethical concerns related to human-wildlife interactions.
5. **Reporting Guidelines**: Explicitly state adherence to relevant reporting guidelines.

With these revisions, the manuscript would meet the criteria for publication in PLOS ONE.

**Recommendation:**

**Major Revisions Required**. The manuscript has potential but requires significant improvements in methodological detail, critical discussion, and adherence to reporting guidelines before it can be accepted for publication.
