# Supplementary material for: Population size, habitat association, and local residents’ attitude towards rock hyrax (Procavia capensis) in Zegie Peninsula, Ethiopia
Source: PLoS One. 2025 Jun 30;20(6):e0323878. doi: 10.1371/journal.pone.0323878 (PMC12208434; doi:10.1371/journal.pone.0323878)
Supplement: Attachment — Submitted filename: Zewdu Kifle_Response to Reviewers.docx [file pone.0323878.s009.docx]

14 April 2025

Ethiopia

Ref.:

PONE-D-24-32415R1

Dear Editor in Chief

**PLOS ONE**

This document constitutes a revised version of our manuscript titled “**Population Size, Habitat Association, and Local Residents’** **Attitudes Toward Rock Hyrax (*Procavia capensis*) in Zegie Peninsula, Ethiopia**” by . Birkie Alehegn and Zewdu Kifle. On 7 March 2025, we received a revision request to make the manuscript acceptable for publication. As per your invitation, we have resubmitted the revised manuscript after thoroughly addressing all concerns and suggestions raised during the peer-review process.

Accordingly, we have addressed all reviewers’ comments in this resubmitted manuscript. Below is a summary of the revisions made:

Sincerely,

Zewdu Kifle,

Corresponding author

**Response for Editor and reviewers**

Thank you for your critical feedback. I have addressed your comments by highlighting revisions in green and marking deleted text (letters, words, phrases, or sentences) in red with strikethrough. Additionally, I have included reviewers’ concerns alongside corresponding justifications within this document. To enhance the manuscript’s quality and clarity, I have also corrected grammatical errors throughout the text.

Thank you!

**Response for Editor**

Thank you! I have revised in detail on the presentation of this manuscript.

**Response for reviewers**

**Reviewer #1:**

Thank you for your important comments.

Line 44: Thank you! I edited the sentence and changed the word ‘harvest’ with ‘crop cultivation’.

Line 62: I added “particularly “

Line 102: Thank you! I deleted this phrase (“the interviewer fully”) when I edited the sentence.

Line 102: I deleted “about”.

Line 103: I deleted “on”.

Line 105: Thank you! I changed “being explained” with “they received a detailed explanation”

Line 114: Thank you for checking the spelling error. I corrected it

Line 121: I changed “are” with “includes”.

Line 144: Thank you! You are right. It is expected that rock hyraxes start their day before 3:00 a.m. However, for this particular research, we collected data starting with this time.

Line 145: Thank you so much!! I edited the time zone with 2:30-5:00 p.m.

Line 175: Thank you! Yes, zero is still the valid observation. However, since it is an extremely outlier value, we excluded it from the analysis.

Line 192-193: Thank you a lot! I corrected this sentence

Line 198-200: Thank you so much again! I corrected the age ratio here too.

Line 224: I edited the phrase.

Line 243: Thank you! I rewrote the sentence.

Line 248: Thank you! I edited the phrase

Line 252: I put this sentence at the end of the paragraph.

Line 259: Thank you! I have edited this word.

Line 261: Thank you! I have edited this word.

Line 262: I changed the word “informed” with “mentioned”.

Line 286: Thank you! I rewrote this sentence.

Line 295: I changed the word “case” with “cause”.

Line 300-301: Thank you! I deleted this sentence from here.

Line 307: I deleted this phrase.

Lie 312: I deleted the phrase “and birth”.

Line 318: I deleted the phrase.

Line 319-320: Thank you! I deleted this phrase.

Line 327: I deleted the word “also”

Line 328: I changed “this might be” with” likely”

Line 335: I added “in the Zegie Peninsula” here.

Line 336: I edited this phrase.

Line 338: Thank you! I deleted the word “develop” and edited it.

Line 342: I preferred the word “behavior” rather as it is

Line 355: I reshuffled this sentence

Line 358: I changed “uneducated” with no formal education

Line 360: Thank you! I edited this sentence.

Line 368: I added “in the Zegie Peninsula” here.

Line 369-370: I deleted this phrase

Line 371: Thank you! I edited this phrase.

Line 381: I preferred the word “fattening” as it is.

Line 381-382: Thank you so much! I rewrote this sentence to emphasis the medical value of rock hyraxes.

Line 383: I deleted “the”.

Line 385: I added “in addition” here.

Line 513: I changed “uneducated” into “no education” with the table.

Line 516: I edited it.

Line 516: I changed “uneducated” into “no education” with the graph.
